# mRNA Analysis of Frameshift Mutations with Stop Codon in the Last Exon: The Case of Hemoglobins Campania [α1 cod95 (−C)] and Sciacca [α1 cod109 (−C)]

**DOI:** 10.3390/biomedicines9101390

**Published:** 2021-10-04

**Authors:** Giovanna Cardiero, Gennaro Musollino, Romeo Prezioso, Giuseppina Lacerra

**Affiliations:** Institute of Genetics and Biophysics “Adriano Buzzati Traverso”, National Research Council, 80131 Naples, Italy; giovanna.cardiero@gmail.com (G.C.); gennaromusollino@gmail.com (G.M.); romeo.prezioso@igb.cnr.it (R.P.)

**Keywords:** α-thalassemia, frameshift, premature termination codon (PTC), unstable α-Hb variants, mRNA quality control, no-go decay, dominant phenotype, Hb Campania or *HBA1*:*c.287delC*, Hb Sciacca or *HBA1:c.328delC*, bioinformatics

## Abstract

An insertion or deletion of a nucleotide (nt) in the penultimate or the last exon can result in a frameshift and premature termination codon (PTC), giving rise to an unstable protein variant, showing a dominant phenotype. We described two α-globin mutants created by the deletion of a nucleotide in the penultimate or the last exon of the α1-globin gene: the *Hb Campania* or α1 cod95 (−C), causing a frameshift resulting in a PTC at codon 102, and the *Hb Sciacca* or α1 cod109 (−C), causing a frameshift and formation of a PTC at codon 133. The carriers showed α-thalassemia alterations (mild microcytosis with normal Hb A2) and lacked hemoglobin variants. The 3D model indicated the α-chain variants’ instability, due to the severe structural alterations with impairment of the chaperone alpha-hemoglobin stabilizing protein (AHSP) interaction. The qualitative and semiquantitative analyses of the α1mRNA from the reticulocytes of carriers highlighted a reduction in the variant cDNAs that constituted 34% (*Hb Campania*) and 15% (*Hb Sciacca*) of the total α1-globin cDNA, respectively. We developed a workflow for the in silico analysis of mechanisms triggering no-go decay, and its results suggested that the reduction in the variant mRNA was likely due to no-go decay caused by the presence of a rare triplet, and, in the case of Hb Sciacca, also by the mRNA’s secondary structure variation. It would be interesting to correlate the phenotype with the quantity of other frameshift mRNA variants, but very few data concerning α- and β-globin variants are available.

## 1. Introduction

The α-thalassemia phenotype is mainly associated with common deletional defects, the formation of which is favored by the high homology between the *HBA1* and *HBA2* globin genes contained in duplication units [1]. We identified about 25% of the α-thalassemia point mutation variants via epidemiological studies in Southern Italy [2]. These point mutations can cause thalassemia by inactivating regulatory sequences, or they can give rise to unstable variants [1,3].

One class of mutants is characterized by the deletion/insertion of a few base pairs (bp), causing frameshift, leading to truncated or elongated protein chains. The frameshift can give rise to globin chains with an altered helix and length, due to reading frame shift, creating a premature termination codon (PTC) or placing a stop codon after the canonical one.

These hemoglobins (Hb) are mostly unstable variants, carrying structural modifications in the α1β1 contact area. The instability is caused mainly by their altered interaction with the chaperone alpha-hemoglobin stabilizing protein (AHSP), which mimics the interaction with the β-chain, but could also be caused by an altered H helix, which is crucial in the building of the heme pocket, or by altered Hb tetramer assembly [3,4,5].

The heterozygote phenotype is very variable. The carriers can be asymptomatic or show only borderline hematological abnormalities. Nevertheless, when the proteolytic mechanisms of erythrocytes are unable to remove all the denatured Hb material, some of these variants may cause mild chronic hemolytic anemia [3]. The frameshift variants are often identified under homozygous or compound heterozygous conditions, with patients showing a more severe phenotype with respect to their genotype, such as Hb H and thalassemia intermedia syndrome [3].

Typically, transcripts that contain PTC undergo nonsense-mediated decay (NMD) to avoid the production of toxic proteins, but when it is located in the last exon, the PTC will escape the surveillance system, and a truncated protein will be translated [6]. The analysis of the stop codons present in the α-globin genes by means of the Virtual Ribosome software, (https://services.healthtech.dtu.dk/service.php?VirtualRibosome-2.0, accessed on 22 July 2021), has shown that in α-globin genes, nine non-in-frame stop codons are present in the coding region (at codons 16, 49, 56, 67, 84, 102, 108, 133, and 137) and three in the 3′UTR (at positions 173, 174 and 176) [7]. The deletions/insertions occurring after codon 84 cause a frameshift that creates a stop codon in the third (last) exon.

In an epidemiological study on the molecular basis of α-thalassemia in Southern Italy, we identified two new variants, each with a deletion of one nucleotide [8,9]. The deletion of C at codon 95 of the α1-globin gene gave rise to the *Hb Campania* gene (α1 cod95 (−C) or *HBA1*:*c.287delC*). This mutation causes a frameshift and, possibly, production of an α-chain variant of 101 amino acids (aa), α95RSTSSS, which we termed Hb Campania. The deletion of C at codon 109 of the α1-globin gene gave rise to the *Hb Sciacca* gene (α1 cod109 (−C) or *HBA1:c.328delC*), with a frameshift causing the formation of a stop codon at 133, possibly resulting in the synthesis of a truncated α-chain of 132 aa [8,9]. Both qualitative and semiquantitative analyses of the mRNA from patients’ reticulocytes revealed a reduction in mutant mRNA. The in silico analysis shed light on factors that could favor the degradation of mRNA.

Finally, we collected the data on frameshift mutants in the second and third exons of the α-globin genes reported in literature, and on two dominant inclusion body β-thal, to compare the phenotypes in relation to the presence of mutated mRNA.

## 2. Materials and Methods

### 2.1. Families

In a study on the molecular basis of α-thalassemia in Southern Italy, the collaborating thalassemia centers selected families and single patients showing a mean corpuscular volume (MCV) lower than 80 fL, which is considered the minimum value for normal Hb A2 and iron status. We determined the molecular basis of α-thalassemia in the 996 families; here, we include the results for 12 carriers belonging to 6 unrelated families living in Sicilia and Campania. A special committee of the Italian Ministry of University and Research approved the study (Decreto n. 250 of 22 June 1999), and two scientists were appointed as supervisors. The participants signed informed consent for the use of blood samples. Ethical approval of the protocol was obtained from the Comitato Etico Università Federico II of Naples (307/2016 and 225/2019).

### 2.2. Hematological Parameters

Hematological parameters, in blood zinc protoporphyrin (*ZPP*), and in serum ferritin, transferrin, and bilirubin (total and indirect) were determined by standard procedures in the collaborating hospitals.

The hemoglobin (Hb) analysis was performed via cation exchange high-performance liquid chromatography (HPLC) (Bio-Rad, Diamat or Variant System Hercules, CA, USA). Heinz body formation and thermal and isopropanol Hb stability tests were performed via standard methods.

### 2.3. DNA Molecular Screening

DNA from white blood cells was purified with the salting-out method [2]. Molecular screenings for the most common α-thalassemia deletions (-α3.7, -α4.2, (α)α5.3) and for the point mutations were carried out by gap-PCR, double gradient–denaturing gradient gel electrophoresis (DG–DGGE), and multiplex amplification refractory mutation system (ARMS), respectively [2,10,11]. Sequencing of the α-globin genes was carried out using an automatic sequencer as previously reported [10]. An ARMS protocol for the direct identification of the mutation at codon 109 was set up and used for the genotyping of the patients [11]. The research activities were carried out inspired by the principles of quality and developing guidelines for the protocols to increase the reliability and reproducibility of the results [12,13].

### 2.4. α-Globin Haplotype

The analyses of the three single nucleotide polymorphisms (SNPs)—RsaI 5′ of the α2-globin gene (rs2541669), α2 + 14 (HBA2:c.−24C > G rs772829778), and α2 + 861 (HBA2:c.*136A > G, rs2685121) for the α-globin haplotype—were performed as previously reported [14,15].

### 2.5. cDNA

mRNA was prepared from reticulocyte-enriched peripheral blood cells and from whole blood, using the Trizol reagent kit, as directed by the manufacturer (Gibco reagent Life Technologies, Inc., Grand Island, NY, USA) [13,14]. In total, 50–200 ng of mRNA was analyzed by reverse transcription PCR (RT-PCR) at a low number of cycles (24 cycles) using rTth DNA polymerase (Roche, Foster City, CA, USA) and the oligonucleotides reported in Table 1. Then, 10 μL of the RT-PCR was separated on a 1.5% agarose gel to identify abnormal fragments.

### 2.6. Semiquantitative mRNA Analysis by Restriction Enzymes

A semiquantitative analysis of the mutated and normal cDNA was carried out via NlaIV and BseDI (Thermo Fisher Scientific Baltics UAB, Vilnius, Lithuania) restriction enzyme (RE) analysis, respectively. The ratio of mRNA synthesized in cells with the two α1-globin alleles was measured in patients that were heterozygous for the Hb variants, taking advantage of the fact that *Hb Campania* and *Hb Sciacca* are associated with the absence of the NlaIV (GGN’NCC) and BseDI (C’CNNGG) restriction sites, respectively. To limit the production of heterodimers, we carried out a first PCR round of 24 cycles and a second round of 1 cycle after increasing the volume of the reaction 20-fold. As a positive control for the enzymatic activity of the RE digestion, and for the normal ratio between the restricted bands, we used the amplicomers from the DNA of WT subjects and Hb Var carriers. To study *Hb Campania*, the DNA of carriers and subjects with WT α1-globin was amplified using the primers A/B in the first round to specifically amplify the α1-globin gene, and the primers C/D in the second round; the cDNA was amplified using the primers E/G in both rounds (Table 1). In the case of *Hb Sciacca*, the amplification was performed using the primers F/G in both rounds for the DNA, and H/G in both rounds for cDNA (Table 1).

The amplicomers were digested, according to the manufacturer’s recommendations, with 10 U of RE, and the restricted bands were size-fractionated in a 3.5% NuSieve 3:1 agarose gel (FMC, Rockland, Maine, USA) at 70 V for 2–3 h. After separation on the NuSieve gel, a semiquantitative analysis of the bands was performed with the Kodak software Carestream MI (Carestream Health, Inc, Rochester, NY, USA). The ratio of undigested/digested bands (that is, mutated/wild type (WT) DNA and cDNA) was obtained as previously reported [13,14,15,16].

### 2.7. Database

All data regarding the families under study and the experimental results were collected anonymously in a database developed on Microsoft Visual Fox 6.0, interfaced with an external software, such as Microsoft Excel or Microsoft Word (Microsoft, Redmond, WA, USA) [17].

### 2.8. In Silico Analysis

The PSIPRED Protein Analysis Workbench was used for the prediction of the secondary structure of the normal and mutant α-chains (http://bioinf.cs.ucl.ac.uk/psipred/, accessed on 12 September 2021) [18].

We evaluated the mutation-induced structural alterations by analyzing the structure of α-chain of human hemoglobin in the complex with AHSP (PDB code 1Y01 and 1Z8U) and in the tetrameric α2β2 structure (PDB code 2HHB), using the programs Yasara (version 20.4.24) (http://www.yasara.org/products.htm, accessed on 12 September 2021) and the Swiss-PdbViewer (version 4.1.0) (www.expasy.org, accessed on 12 September 2021) [19,20,21,22] (Figures S1 and S2).

The Virtual Ribosome web site was used to identify the stop codon in the *HBA1* cDNA (https://services.healthtech.dtu.dk/service.php?VirtualRibosome-2.0, accessed on 22 July 2021) [7].

The programs SIFT (Sorting intolerant from tolerant) (https://sift.bii.a-star.edu.sg/www/SIFT_indels2.html, accessed on 18 June 2021) (Figure S3), MutationTaster (http://www.mutationtaster.org/, accessed on 21 June 2021) (Figure S4), and Splice site prediction (by Neural Network software, https://www.fruitfly.org/seq_tools/splice.html, accessed on 30 June 2021) (Figure S5) were used to verify the activation of alternative splicing, ascertain the lengths of abnormal proteins (Figure S6), and determine whether the NMD could trigger the mRNA quality control mechanism [23,24,25]. The Expasy bioinformatic resource portal was queried for the in-frame translation (Figure S7) and to acquire the protein sequences (https://web.expasy.org/translate/, accessed on 21 June 2021) and amino acid compositions of the variant and WT proteins (https://web.expasy.org/protparam/, accessed on 22 June 2021) (Figure S8) [26]. The CAIcal Server (http://genomes.urv.es/CAIcal/, accessed on 23 June 2021) (Figure S9) and the Sequence manipulation suite (SMS, https://www.bioinformatics.org/sms2/codon_usage.html, accessed on 22 July 2021) were queried for the codon usage and to compare the mutant and WT mRNA [27,28]. The Kazusa software (https://www.kazusa.or.jp/codon/cgi-bin/showcodon.cgi?species=9606, accessed on 21 June 2021) was used to determine the frequency of codon usage in the Homo sapiens and human target tissue (Figure S10). The mRNA secondary structure was predicted, using the RNAfold web server (http://rna.tbi.univie.ac.at/cgi-bin/RNAWebSuite/RNAfold.cgi, accessed on 16 June 2021) [29].

## 3. Results

### 3.1. Hb Campania [α1 cod95 (−C)]

#### 3.1.1. Molecular Characterization and cDNA Analysis

**Figure 1:**
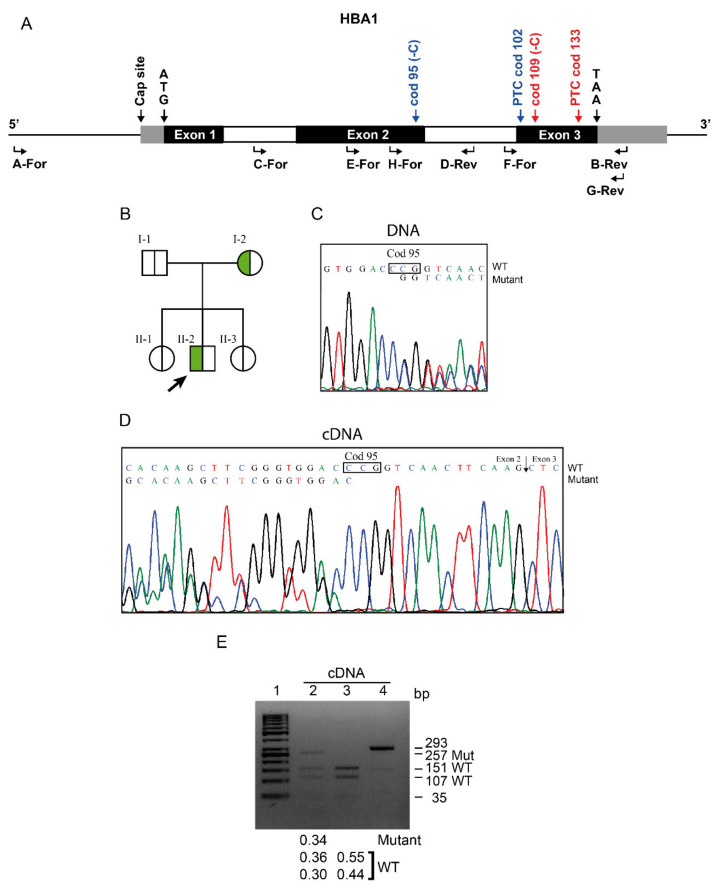
Molecular characterization and cDNA analysis of *Hb Campania*. (**A**) Scheme of the functional structure of the α1-globin gene (HBA1), indicating the position of cod95 (−C) and cod109 (−C) with their relative premature termination codon (PTC). The gray rectangles indicate the 5′ and 3′ UTR regions, the white rectangles the introns. The positions and orientations of the primers used for the molecular characterization are indicated with arrows placed under the gene. (**B**) Pedigree of the family. The arrow indicates the proband; green indicates the carriers of *Hb Campania*. (**C**,**D**) α1-globin gDNA (**C**) and cDNA (**D**) sequences of a carrier of *Hb Campania*. (**E**) The cDNA amplicomers of 293 bp, digested by the restriction enzyme NlaIV, and separated on a 3.5% NuSieve 3:1 agarose gel. Lane 1: 50 bp ladder; Lane 2: cDNA of the *Hb Campania* carrier; Lane 3: cDNA of the control subject; Lane 4: undigested cDNA sample. The fragments’ lengths are reported on the right. The *Hb Campania* eliminates the NlaIV restriction site GGA’CCC, generating an anomalous longer cDNA band of 257 bp, corresponding respectively to the sum of the two WT-specific bands of 151 and 107 bp, minus the deleted cytidine base. The relative amounts of the longer abnormal band and the WT-bands are reported in the lower section.

The new point mutation, giving rise to the *Hb Campania* allele, or α1 cod95 (−C), was identified in a family from Naples (Figure 1A,B). The two carriers showed mild α-thalassemia hematological alterations with reductions in the mean corpuscular volume (MCV; 76 and 80 fL) and mean corpuscular hemoglobin (MCH; 24.6 and 23.6 pg). These patients’ serum iron, ferritin, transferrin, total bilirubin, and reticulocytes were within the normal ranges. Abnormal hemoglobin or globin chains were not detected via electrophoresis or ion-exchange HPLC. The Hb A2% levels were in the normal range (Table 2).

Screening for the α-thalassemia deletions gave negative results, and the sequencing analysis of the α1- and α2-globin genes only revealed a cytidine deletion at codon 95 of the α1-globin gene. The mutation was confirmed by sequencing in the other members of the family (Figure 1C). The α1 cod95 (−C) mutation caused a frameshift and, possibly, production of an α-chain variant of 101 aa, α95RSTSSS.

The RT-PCR and the sequencing of α1-globin cDNA, performed on mRNA purified from reticulocytes from fresh blood, indicated a frameshift at codon 95, but this mutated sequence exhibited base peaks much smaller than those of the WT sequence (Figure 1D).

To quantify the mutated mRNA, we performed a semiquantitative analysis by digestion with the NlaIV RE, for which the mutation eliminates a restriction site, as shown in Figure 1E. The DNA digestion confirmed the presence, in the carriers, of an anomalous band of 285 bp, specific to the *Hb Campania.* The relative amount of this anomalous band was comparable (0.50) to the sum of the relative amount of the two WT bands (225 and 61 bp) on DNA of the *Hb Campania* heterozygote, indicating the presence of the WT and mutant alleles (Figure S11A). Otherwise, the digestion on cDNA from the reticulocytes of the carrier indicated that the relative amount of the anomalous 257 bp band, specific to *Hb Campania*, was 0.34 respect to the total α1-globin cDNA, as shown in Figure 1E. These data confirmed a consistent reduction in *Hb Campania* cDNA.

#### 3.1.2. 3D Modeling

To define the effect of the frameshift on the protein stability, a 3D model of the Hb Campania α-chain was created by means of SWISS-MODEL. The structures of the WT α-chain interacting with AHSP (1Y01, 1Z8U) and in the Hb tetramer (2HHB) were used to fit the Hb Campania α-chain [21,22]. The analysis of the 3D models (Figure S1A–F) highlighted two aspects: the Hb Campania is superimposed with the WT α-chain also in the modified C-terminal region, where the change of the 6 aa is present; an empty region is evident in the variant chain for the absence of the two prominent G and H helices.

The 6 mutated aa are posed in the central cavity; these 6 aa, in the WT α-chain, are involved in the α1β1 contacts and three of them in the heme contact (cod 97, 98, 101) [30]. The frameshift introduces 1 polar and 5 small non-polar aa instead of 2 polar, 1 small non-polar, 2 hydrophobic, and 1 aromatic aa (Figure S2). These differences most likely cause disturbances on the functions of these 6 aa.

**Figure 2:**
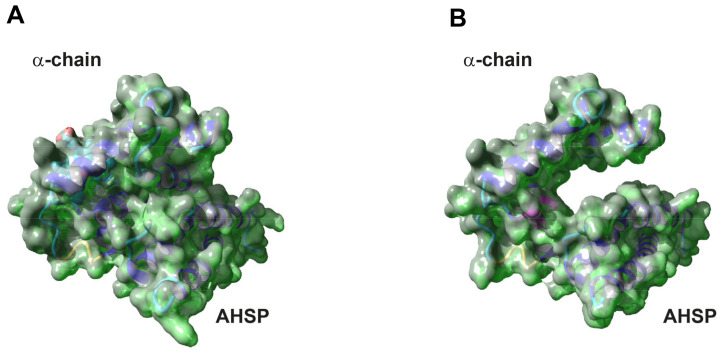
The 3D surface model of the Hb Campania. (**A**) The 3D surface model of the WT α-chain and of the Hb Campania (**B**) in complex with AHSP (PDB code 1Z8U).

The generation of the 3D surface models of the WT and Hb Campania α-chains in complex with AHSP better highlighted the presence of a big cavity in the Hb Campania that compromises multiple functions: the central cavity is exposed; the interaction with the heme group is not stabilized; the interactions with AHSP are insufficient to stabilize the α-chain (Figure 2A,B) [5,30,31]. The 3D model of Hb Campania in the Hb tetramer is equally compromised by the absence of the two G and H helices important also in the interaction between the α- and β-chains (Figure S1D–F).

The analyses of the 3D models indicate that the Hb Campania is unstable.

#### 3.1.3. In Silico Analyses

To investigate the possible causes of the reduction in mRNA, we analyzed, in silico, alternative splicing, mRNA structure, and codon usage.

The analysis of alternative splicing gave negative results, suggesting that the deletion cod95 (−C) has no impact on the consensus sequence for splicing (Figures S3–S5). The RT-PCR amplification of the total length α1-globin mRNA confirmed the absence of an anomalous fragment and, thus, of alternative splicing (data not shown).

**Figure 3:**
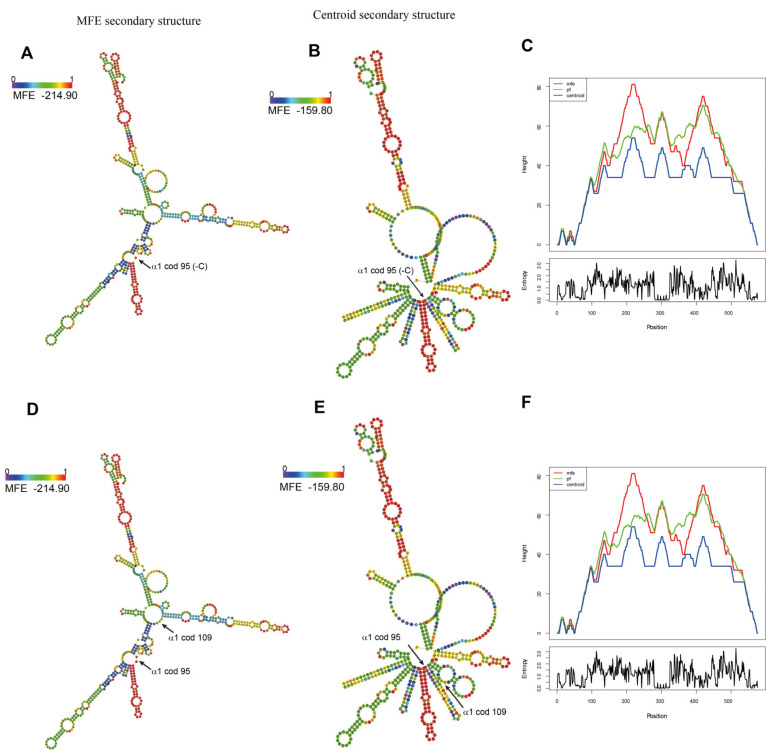
Secondary structure of α1-globin mRNAs predicted by the RNAfold web server. (**A**,**B**,**C**) α1 cod95 (−C) mRNA. (**D**,**E**,**F**) WT α1-globin mRNA. (**A**,**D**) Minimum free energy (MFE) secondary structure. (**B**,**E**) Centroid secondary structure with the corresponding values. (**C**,**F**) Mountain plot representation of the MFE structure for each α1-globin mRNA. The values of α1 cod95 (−C) were comparable with the values of WT α1 mRNA.

The analysis of alterations in the mRNA’s structural conformation indicated that the structure and stackable minimum free energy (MFE) of *Hb Campania* (Figure 3A–C) were comparable to those of WT mRNA (Figure 3D–F); therefore, this mechanism seems uninvolved in the activation of mRNA quality control [32,33,34].

**Figure 4:**
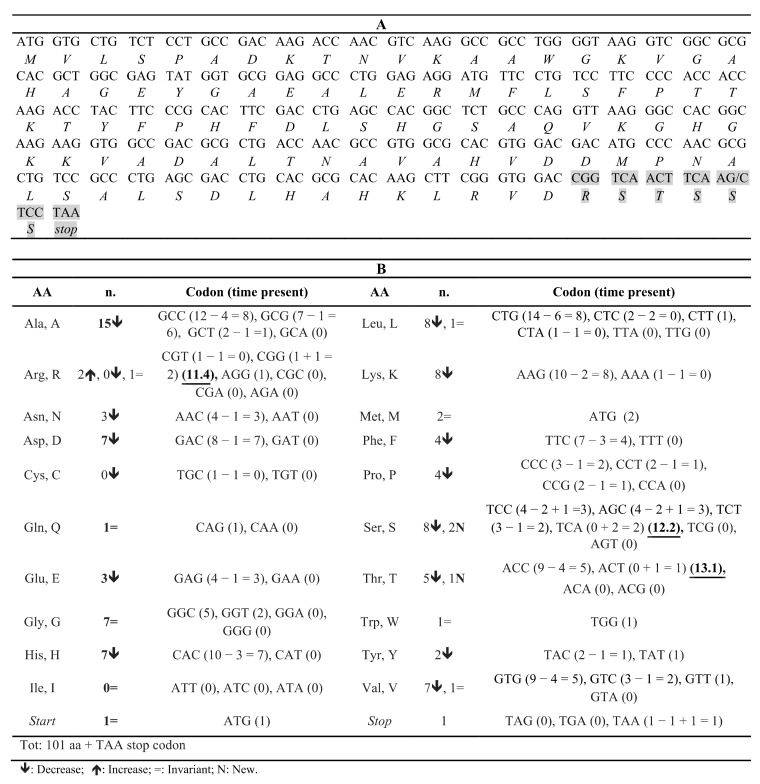
(**A**) Nucleotide triplets coding the *Hb Campania* (α1 cod95 (−C)) mRNA, from the ATG to the stop codon at position 102; below, the corresponding amino acids present in this α-globin chain variant. The changes in amino acids following the frameshift after cod95 are shown in gray. The symbol/indicates the position of the exon2/3 boundary. (**B**) Types of amino acids present in the Hb Campania α-globin chain, the corresponding coding triplet, and the number of appearances in the *Hb Campania* mRNA. The numbers underlined and in bold indicate the codon usage frequencies.

The in silico analyses of amino acid composition and codon usage in Hb Campania highlighted several changes, caused mainly by the considerable reduction in length: 25 codons were used fewer times, 1 codon was used more frequently, and there were 2 codons used for the first time (Figure 4, Figures S6–S8). For the latter two classes, the analysis of human codon usage showed high frequencies for the three codons CGG (1 + 1 = 2) (11.4), TCA (0 + 2 = 2) (12.2), and ACT (0 + 1 = 1) (13.1) (Figure 4, Figures S9 and S10). Moreover, the codon CGG was absent in the mRNA encoding the β-chain and present once in the one encoding the α-chain; the codon TCA was absent in both these mRNAs, while ACT was present three times in the β-globin mRNA and absent in the α-globin mRNA. These data highlight that in reticulocytes, where the synthesis of globin chains is the highest, the two tRNAs for the CGG and TCA codons may be less common, and therefore, the synthesis of the variant globin chain may be slower (Figure 4) [35]. In silico analyses of the codon usage both in red blood cells and in the WT hemoglobins confirmed the low frequencies of CGG and TCA in the target tissue (Figure S10B,C).

### 3.2. Hb Sciacca [α1 cod109 (−C)]

#### 3.2.1. Molecular Characterization and cDNA Analysis

In 10 patients from 5 apparently unrelated families originating from Sicily, we identified a rare α-globin chain variant created by the deletion of C at cod109 of the α1-globin gene, named *Hb Sciacca* or HBA1: c.328delC [5,7].

The probands showed normal iron balance, no clinical symptoms and were sent to us for evaluation of their mild microcytic anemia. All 10 carriers showed α-thalassemia alterations (microcytosis with normal Hb A2). Their MCV ranged from 73.2 fL to 81.8 fL, and their MCH from 23.8 pg to 27.1 pg. Their Hb A2 % level was in the normal range (Table 3).

The proband II.2 of family B was reexamined and displayed reticulocytes, indirect bilirubin, haptoglobin, LDH, and pink test results within the normal range as well as the absence of Heinz bodies (Table 3). No instability test could be performed on fresh blood, but the analysis in our laboratory after shipping, was normal. All these data indicated the absence of hemolytic processes. The HPLC and electrophoresis carried out on the hemolysate revealed no Hb Sciacca.

**Figure 5:**
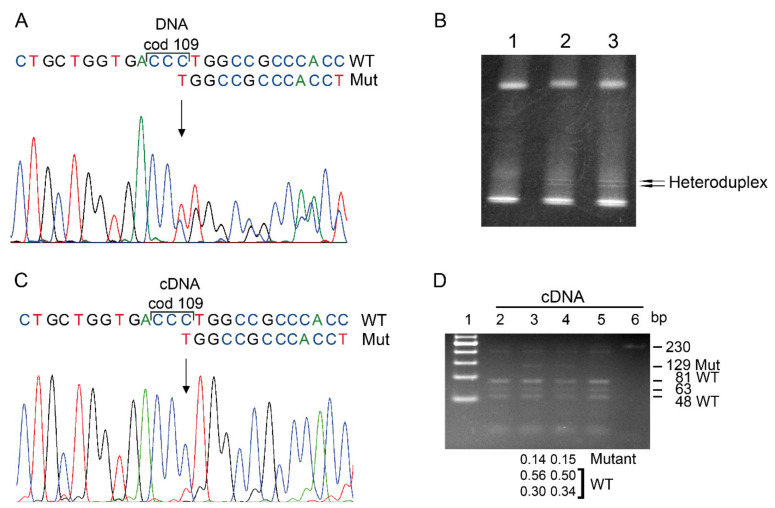
Molecular characterization and cDNA analysis of *Hb Sciacca*. (**A**) α1-globin gDNA sequence of an *Hb Sciacca* carrier. (**B**) Denaturing gradient gel electrophoresis (DGGE) of amplicomer III of the α-globin genes containing codon 109. Lane 1: subject with WT α1-globin; Lanes 2 and 3: *Hb Sciacca* heterozygotes. (**C**) α1-globin cDNA sequence of an *Hb Sciacca* carrier. (**D**) The cDNA amplicomers of 230 bp, digested with the restriction enzyme BseDI and separated on a 3.5% NuSieve 3:1 agarose gel. Lane 1: 50 bp ladder; Lanes 2 and 5: cDNA of subjects with WT α1-globin; Lanes 3 and 4: cDNA of the *Hb Sciacca* heterozygotes; Lane 6: undigested cDNA sample. The *Hb Sciacca* eliminates the BseDI restriction site C’CCTGG, generating an anomalous longer cDNA band of 129 bp, corresponding to the sum of the two WT-specific bands of 81 and 48 bp, minus the deleted cytidine base. The fragments’ lengths are reported on the right. The relative quantities of longer abnormal bands and WT bands are reported in the lower section.

Gap-PCR excluded the presence of any of the following α-thalassemia alleles: -α3.7, -α4.2, and (α)α5.3. The double gradient–denaturing gradient gel electrophoresis (DG-DGGE) of five DNA PCR amplicomers, spanning the α1- and α2-globin genes, detected an abnormal pattern in their third exons (Figure 5B). The sequencing of anomalous amplicomers identified the rare mutation α1 cod109 (−C), which causes a frameshift (Figure 5A) and modifies the C-terminal sequence, creating an α-chain variant of 132 amino acids: α109WPPTSPPSSPLRCTPPWTSSWLL (Figures S6–S8). No other mutation was identified via the sequencing of the α1- and α2-globin genes. The mutation was confirmed in all members of the families, using the amplification refractory mutation system (ARMS). Analysis of the three SNPs RsaI(+), +14(–), and +861(–) identified the same α-globin haplotype in each of the five families with *Hb Sciacca*.

A qualitative and semiquantitative analysis on the α-globin mRNA was performed to evaluate its level of expression. RT-PCR and cDNA sequencing performed on the mRNA from reticulocytes in blood identified a frameshift at cod109, but the variant sequence α1 cod109 (−C) showed base peaks much smaller than those of the WT sequence (Figure 5C). In order to quantify the mutated mRNA, we performed a semiquantitative analysis by digestion with the BseDI restriction enzyme, for which the mutation eliminates a restriction site. The DNA digestion confirmed, in the carriers, an anomalous 93 bp band, specific to the *Hb Sciacca*. The relative amount of these anomalous bands constituted 54% and 58% of the total α1-globin gene bands in the two carriers. These data confirmed that both the alleles *Hb Sciacca* and WT α1-globin gene are present in the carriers (Figure S11B).

Otherwise, the digestion on cDNA from the reticulocytes of the two carriers indicated that the relative amount of the anomalous 129 bp bands, specific to *Hb Sciacca*, were 14% and 15% of the total α1-globin mRNA as shown in Figure 5D.

These data indicated an unexpected consistent reduction in *Hb Sciacca* cDNA.

#### 3.2.2. 3D Modeling 

To define the effect of the frameshift on the protein stability a 3D model of the Hb Sciacca α-chain was created (Figure S1G–L) as already reported for Hb Campania.

The analysis of the composition of the 23 mutated aa indicated a marked reduction in the polar (4% instead of 21%) and hydrophobic aa (13% instead of 24%) and an increase in small non-polar (65% instead of 45%) and aromatic aa (17% instead of 9%) (Figure S2). This consistent change in the number and type of aa between the WT α-chain and the Hb Sciacca suggests a negative impact of the variations on the inter- and intra-chains interactions.

**Figure 6:**
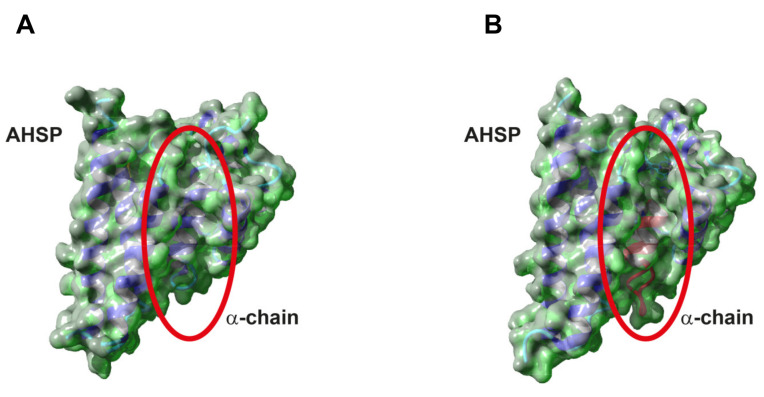
The 3D surface model of the Hb Sciacca. (**A**) The 3D surface model of the WT α-chain and of the Hb Sciacca (**B**) in complex with AHSP (PDB code 1Z8U). The red circles indicate the position of 23 mutated aa (in magenta) and the small cavity for the absence of the H helix in the Hb Sciacca (**B**) and the corresponding position in the WT (**A**).

The 3D superimposed model highlights that the Hb Sciacca retains almost the same tertiary structure up to the F helix, but overall, it is evident the presence of a longer mutated GH non-helix region of 11 aa (instead of 6 aa) at cod113-123, and of a shorter mutated H helix of only 7 aa (instead of 21 aa) (Figure S1G–L). The 3D surface model of the Hb Sciacca α-chain confirmed and highlighted its structure variation (Figure 6A,B).

The H helix in the WT α-chain has important roles; its shortening and variation in the composition, observed in the Hb Sciacca, leads to the alteration and destabilization of the tertiary structure. The Hb Sciacca impairs the correct formation of the central cavity and of the heme pocket—for the modifications at cod 129 Leu>Pro (H2) and the absence of the Leu 136, both involved in the heme contact—and it impairs also the correct interaction with AHSP for the mutation, among others, at cod 117 (G1) Phe>Pro [3,4,5,30,31].

The tertiary structure is also modified for the presence of a bulky non-helix GH region, which, in the WT α-chain, is in an external position and involved in the α1β1 contacts, while, in the Hb Sciacca, it most likely creates interference both in the interaction with AHSP and with the β-chain (Figure 6A,B).

The analyses of the 3D models indicate that the Hb Sciacca is unstable.

#### 3.2.3. In Silico Analyses

To understand the causes of the reduction of the *Hb Sciacca* mRNA, we performed an in silico analysis to highlight the mechanisms that could trigger the mRNA quality control decay.

The prediction of variations in the splicing site (https://www.fruitfly.org/seq_tools/splice.html, accessed on 30 June 2021), SIFT (accessed on 18 June 2021) and MutationTaster (accessed on 21 June 2021), Figures S3–S5 indicated that the mutation does not change the splicing score (0.02) of the cryptic splicing sequence (cctgctgGTgaccct → cctgctgGTgacctg) that normally occurs between codons 104 and 109. The RT-PCR amplification of the total length α1-globin mRNA confirmed the absence of anomalous fragments and thus, of alternative splicing.

**Figure 7:**
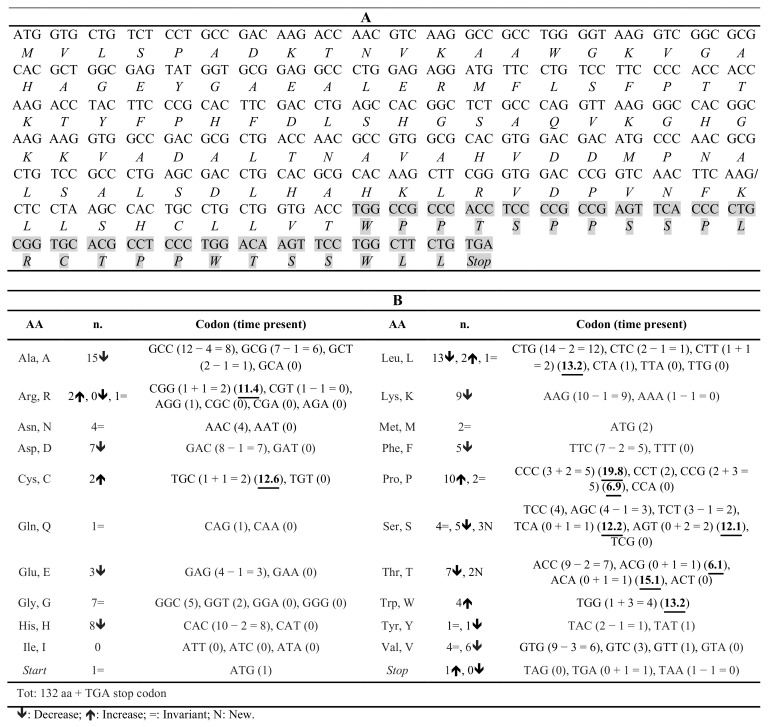
(**A**) Nucleotide triplets coding the *Hb Sciacca* mRNA, from the ATG to the stop codon at position 132; below, the corresponding amino acids present in this α-globin chain variant. The changes in Hb Sciacca amino acids (aa) following the frameshift from cod109 are marked in gray. The symbol / indicates the position of the exon2/3 boundary. (**B**) Types of aa present in the Hb Sciacca α-globin chain, the corresponding coding triplet, and the number of occurrences in the *Hb Sciacca* mRNA. The numbers underlined and in bold indicate the codon usage frequencies.

The analysis of amino acid composition (Figures S6–S8) and codon usage (Figures S9 and S10) in the Hb Sciacca variant indicated several changes: 16 codons were used fewer times, 6 codons more frequently, and there 5 were novel codons. Among the last two classes, two changes are noteworthy: the CCG codon was present five times instead of two, and ACG appeared once (Figure 7). Data on codon usage in humans (https://www.kazusa.or.jp/codon/cgi-bin/showcodon.cgi?species=9606, accessed on 21 June 2021) show that CCG and ACG are rarely used, fifth (6.9) and third (6.1) least frequent codons, respectively, excluding the stop codons (Figure S10A). The analysis of human codon usage both in red blood cells and in WT hemoglobin confirmed low frequencies of these two codons (Figure S10B,C). These data suggest that the slowing down of the synthesis of Hb Sciacca, due to the presence of CCG and ACG, could activate the no-go decay [32,33].

**Figure 8:**
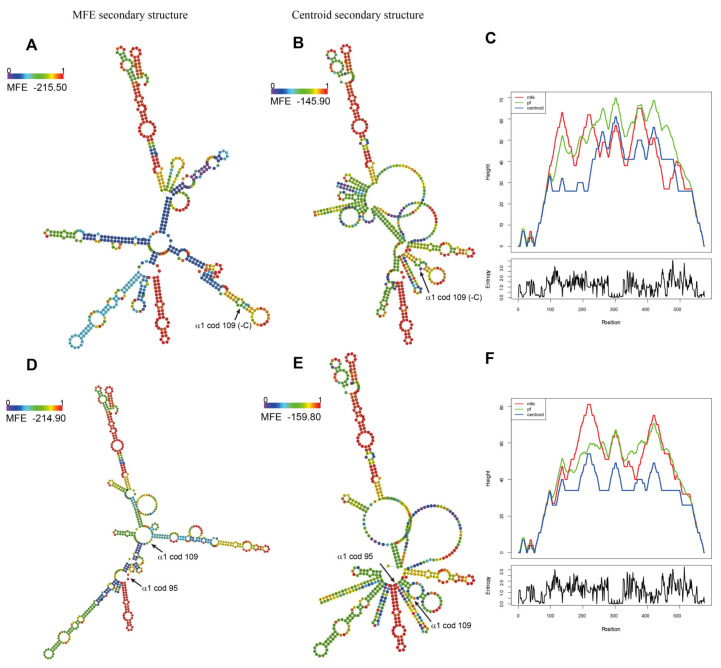
Secondary structure of α1-globin mRNAs predicted by the RNAfold web server. (**A**,**B**,**C**) α1 cod109 (−C) mRNA. (**D**,**E**,**F**) WT α1-globin mRNA. (**A**,**D**) Minimum free energy (MFE) secondary structure. (**B**,**E**) Centroid secondary structure with the corresponding values. (**C**,**F**) Mountain plot representation of the MFE structure for each α1-globin mRNA. The values of α1 cod109 (−C) presented a small variation in free energy of the secondary structure. The centroid secondary structure of α1 cod109 (−C) presented a variation in free energy. The presence of differences in the secondary structure conformation of the α1 cod109 (−C) mRNA could alter the access of the ribosomal apparatus.

We also investigated the alterations in mRNA structural conformation that could stall the ribosomes during translation [32,33,34]. Hb Sciacca showed only a minimal variation in MFE (−215.50 instead of −214.90) (Figure 8A–C) respect to the WT α-globin mRNA (Figure 8D–F), but a consistent alteration in the secondary structure, with an increase in the number of lateral branches that could interfere with the sliding of the ribosome.

These in silico analyses identified two mechanisms that could stall translation—the alteration of mRNA stability and structure, and the presence of codons that are rarely represented in the cell. These factors could induce no-go decay.

## 4. Discussion

The frameshift mutations in the third exons of the α- and β-globin genes generate variants with altered structures and lengths, which, in most cases, give rise to hyper-unstable hemoglobin [1,3]. Mutants of this type have two peculiar characteristics: one related to the phenotype and the other to the mRNA quality control.

Carriers of these variants, mainly of mutations in the β-globin gene, can exhibit the dominant form of thalassemia, resulting in a thalassemia intermedia phenotype in individuals who have inherited only a single copy of the abnormal globin gene [1]. The dominant phenotype associated with frameshift variants has also been identified in other hereditary diseases [36,37,38].

The second peculiarity is that the premature termination codon (PTC) generated by the frameshift, and the change in reading frame it imposes, is not recognized by the nonsense-mediated decay (NMD) system when in the third (last) exon [4,39].

### 4.1. Hb Campania [α1 cod95 (−C)]

In two members of a family from Campania, we identified a novel cytidine deletion at cod95, associated with a mild α-thalassemia phenotype (Figure 1B,C). The mutation has not been identified in other Italian or Mediterranean areas.

This novel α1 cod95 (−C) mutation results in a frameshift that causes the variation in 6 aa at position cod96-101 and a PTC at position 102 (Figure 1A) with the absence of the last 41 aa. *Hb Campania* is the first discovered deletion of one bp that causes a frameshift after cod84 and the formation of a truncated α-globin chain at cod 102.

This variant is expected to consist of 101 aa and has an altered C-terminal aa sequence, α95RSTSSS (Figure 4, Figures S4 and S6–S8). The absence of the 41 residues, corresponding to the G and H helices, most likely impairs several functions.

The G and H helices play an important role in the intra-chain bonds, such as the formation of the central cavity and of the heme pocket, but also in the inter-chains interactions, through the specific recognition of AHSP and α1β1 contacts [5,30,31].

The analysis of the 3D models of the Hb Campania indicated the presence of a large cavity due to the absence of the G and H helices, which compromise the formation of the correct structure and function of the variant α-chain. In particular, the inability of the Hb Campania α-chain to interact with the AHSP chaperone causes strong instability (Figure 2, Figure S1A–F). The mutant α-chains impairing the interactions with AHSP are usually characterized by a rapid degradation and for this reason often are not identified [15,16]. The Hb Campania has not been identified in the peripheral blood of patients, and this confirms that the variant chain Hb Campania is unstable and could be rapidly degraded and not involved in the formation of the hemoglobin tetramer.

The sequencing analysis of mRNA from reticulocytes of the carriers indicated that the frameshift variant showed a smaller peak than the one of the WT, and the semiquantitative analysis confirmed a reduction in the mutant mRNA that was about 34% of the total α1-globin cDNA (Figure 1D,E). This result adds another reason that explains the absence, in the peripheral blood, of the abnormal Hb Campania, which can only be synthesized at low quantities.

The characterization of the NMD pathway has defined many parameters of the decay process. In particular, in mammalian cells, a termination codon is usually “premature” if it is located at more than 50–54 nucleotides downstream from the final exon–exon junction [35,40,41]. The cod95 (−C) mutation generates a stop codon at a position +5 from the exon2–exon3 junction, which is a much smaller distance than the minimum of 50–54 nt required to activate the NMD, as was also confirmed by the SIFT analysis (Figure S3). This suggests that other mechanisms are involved in the reduction in this mRNA. The reduction in *Hb Campania* mRNA could be a consequence of the activation of one of the several distinct mechanisms that control the quality of mRNA and proteins during translation at the ribosome, which reduce the toxic effects of aberrant proteins causing many human diseases [39,40]. Analysis of the different mRNA control pathways showed that, in the case of *Hb Campania*, the no-go decay mechanism could be active. The no-go decay mechanism degrades mRNAs that are stalled in the translation elongation complexes as a result of either the specific features of the nascent peptides, strong secondary structures in mRNA physically blocking the translation machinery, or a rare codon repeat causing the codon site to be unoccupied for too long [33,34].

The in silico analysis excluded the presence of *Hb Campania* mRNA alterations that could induce the stall of ribosomes and the activation of mRNA quality control (Figure 3A–C) [32,33,34]. On the contrary, the in silico analyses of the amino acid composition (Figure 4, Figures S6–S8) and of the codon usage (Figures S9 and S10) in the *Hb Campania* mRNA highlighted several changes, in particular the introduction of two codons (CGG and TCA) for which the corresponding tRNAs shown low frequencies in the red blood cells. These two rare tRNAs most likely induce a slowdown in the synthesis of *Hb Campania* mRNA, with consequent activation of the no-go decay.

### 4.2. Hb Sciacca [α1 cod109 (−C)]

In an epidemiological study on the molecular basis of α-thalassemia in Southern Italy, in 5 unrelated families, we identified 10 carriers of the novel mutation α1 cod109 (−C), and named it *Hb Sciacca* because it was first identified in a carrier from Sciacca village. The five families all originated from West Sicily.

This novel α1 cod109 (−C) mutation results in a frameshift, causing an altered amino acid sequence in the next 22/23 amino acids, with the inclusion of 7 prolines, and a PTC at position 133 (Figure 1A). The α-chain variant is expected to consist of 132 amino acids and end by the sequence α109WPPTSPPSSPLRCTPPWTSSWLL (Figure 7).

The analysis of the Hb Sciacca superimposed 3D models revealed two anomalies: the presence of a bulky GH non-helix segment longer than the normal, and a shorter mutated H helix causing an empty region (Figure 6A–B, Figure S1G–L). These modifications cause severe structural alterations that compromise important functions as the formation of the central cavity and of the heme pocket, the interaction with AHSP and the β-chain. All these data indicated that the severe structural distortion in the Hb Sciacca could impair the interactions with AHSP, inducing instability and a rapid degradation, and preventing the formation of the Hb tetramer. The absence of the Hb Sciacca in the patients’ peripheral blood supports this hypothesis.

The mutation was also identified in a Kurdish family and in four unrelated patients of Egyptian origin and was associated, in all the cases, with a mild reduction in MCV (72 fL) and MCH (24.3 pg). The authors of these studies described a frameshift mutation in the α1-globin gene at codon 108 (ACC-AC) and assigned to the same mutation two HGVS names HBA1:c.327delC and HBA1:c.328delC [42,43]. This mutation was detected in families from West Sicily, located close to the sea, but also in African and Jewish populations. These findings suggest that the mutation entered this region via a Mediterranean population that settled in West Sicily, or that the mutation originated in Sicily and then spread to other territories as a result of migration. Based on the identification of the same haplotype in all families, we demonstrated unique origin of *Hb Sciacca* in Sicily. It would be interesting to analyze the same SNPs in African and Jewish carriers in order to determine whether the origin is the same between the two continents.

The codons 108 (ACC) and 109 (CTG) are characterized by a CCC strand between them. Given that it is not possible to define the exact position of the cytidine deletion, and that codon 108 remains invariant in any case (ACC), we assume that the deletion affects codon 109, where the frameshift begins; therefore, we have defined the mutation as α1 cod109 (CTG>-TG) or HBA1: c.328delC [6,7,8].

To study in depth the pathophysiology of this variant and the effect of the frameshift on the phenotype, we studied the effects of the mutation on the mRNA. The sequencing revealed that the variant cDNA had smaller peaks compared with the WT one, and the semiquantitative analysis confirmed that the variant mRNA constituted only 14% of the total α1-globin cDNA (Figure 5D).

The in silico and the molecular analysis excluded the activation of an alternative splicing.

*Hb Sciacca* create a premature termination in the third exon of the α-globin gene, caused by a frameshift. In the α-globin gene, two other stop codon mutants were described; they lead to an early termination of translation due to a nonsense mutation—at cod116 and cod127 (Table 4) [44,45]. The abnormal hemoglobin created by the cod116 mutation was not found in peripheral blood, while a quantitative analysis of cod116 mRNA identified it at quantities equal to those of normal mRNA [44]. The mutant at cod127 was not studied at mRNA-level, and the variant chain was not identified [45].

These observations confirm that the quality control mechanism NMD is not activated by mutations creating premature termination codons in the third exon, which produce a stable messenger RNA (mRNA) that is available for direct synthesis of truncated polypeptides [4,39].

To investigate the possible causes that could activate the no-go decay and the reduction in mRNA, we analyzed in silico codon usage and mRNA structure (Figure 7 and Figure 8). These in silico analyses identified two mechanisms that could stall translation: (a) a consistent alteration of *Hb Sciacca* mRNA structure, showing several lateral branches (Figure 8A–C); (b) a consistent change in the number and types of aa—as consequence of the frameshift—in particular, the introduction of two codons (CCG and ACG) rarely represented in the cell (Figure 7). These factors could cause stall of the ribosomes during translation inducing no-go decay [32,33,34].

### 4.3. mRNA Variant in Globin Mutants

To determine whether quality control mechanisms other than the NMD are usually activated in frameshift mutants, we proceeded in two directions: (a) we analyzed a dominant β-thal gene with a frameshift in the third exon via an mRNA analysis; (b) we sought all the frameshift mutations in the third exon of the α-globin gene, in order to collect data on mRNA that could be correlated with the hematological phenotype and with the mechanism of mRNA quality control (Table 4).

In β-globin genes—which are present as a single copy, contrarily to the duplicated α-globin genes—the frameshift in the third exon produces a dominant phenotype, as was well-studied by Thein [4,46]. Two dominant mutants offer information regarding the anomalous β-globin mRNA. We reviewed the available data on the dominant mutated β-globin codons 128/129 (−4, −GCTG; +5, +CCACA) and codons 132–135 (−11, −AAAGTGGTGGC) (HGVS HBB: c.[385_388delinsCCACA; 397_407delAAAGTGGTGGC]) that cause a frameshift reading through to codon 153, which should result in the synthesis of a variant β-globin that is seven residues longer than the WT. However, the author reports that it was not possible to isolate the variant β-chains, and that the total cytoplasmic RNA of the propositus showed a normal α/βmRNA ratio of 1.4 [1]. Furthermore, mRNA analysis of the mild dominant β-codon 120/121 (+A), generating an abnormal β-mRNA with a new stop codon at codon 139 (TAA), showed an α/β ratio of 5.18 (4.44 in the control), indicating that the variant β-globin mRNA was stable, and that neither NMD nor other mRNA quality control mechanisms were active for this mutant [47].

These observations indicate that the variations in codon usage and alterations in mRNA structure that are plausible in these complex β-globin mutants cannot always activate mRNA degradation through quality control mechanisms.

Regarding the mutations in the α-globin genes, five variants were described in close proximity to the Hb Sciacca, and they are characterized by a frameshift because of a deletion or insertion generating a stop codon at position 132, leading to almost the same 3′ protein structure. They are Hb Lynwood (α2 cod107 (−T) or HBA2: c.323delT) [49]; α1 cod111–115 (−13bp) or HBA1: c.333_345delCGCCCACCTCCCC [43]; α2 cod114 (−C) or HBA2: c.345delC [50]; α2 cod115 (+CC) or HBA2: c.343_344insCC [51] (Table 4). All these variants result in a disturbed amino acid sequence between the frameshift codon and the premature stop codon at position 133. The relevant helices G and H alter the aa sequence via the inclusion of several prolines (from 5 to 8), which are most likely very disruptive to the tertiary structure and contribute to the instability of the proteins. Additionally, in the case of Hb Sciacca, the aa sequence that is altered from position G16 through the following 22 amino acids (containing seven proline residues) could result in a very disruptive tertiary structure, altering the interactions with the alpha-hemoglobin stabilizing protein (AHSP) and the β-chain.

In the third exon, other unstable variants were described; specifically, four variants create a very long α-chain: α2 cod90-93 (−8bp) or HBA2: c.272_279delAGCTTCGG (stop at codon 170) [48], α2 cod116–119 (−11bp) (stop at codon 166) [52]; Hb Pak Num Po (stop at codon 175) [55]; Hb Wayne (stop at codon 147) [57]. Patients compound heterozygous for these variants and an α0-thal or α+ -thal mutation are characterized by more severe phenotypes, including transfusion dependence.

The last four mutations generate a stop codon close to the frameshift: Hb Hamilton Hill or α2 cod129 (H12) (−C) (stop at cod133) [53]; Hb Fez or α1 cod131 (H14) (−T) (stop at cod133) [54]; Hb Aalesund or α2 cod133-135 (−7bp) (stop at cod137) [56]; and Hb Senlis or α1 cod134 (H17) (−C) (stop at cod137) [54]. These variants, with the exception of Hb Hamilton Hill, present with chronic hemolytic anemia (CHA) or a balanced CHA. This may be due to the few chain variations in the H helix that still favor the interaction with the AHSP chaperone and with the β-chain, but which create severe instability with the consequent CHA.

Our search for data on mRNA analyses of all these α-globin frameshift variants produced no meaningful information because, to the best of our knowledge, other authors did not carry out analyses on the mRNA from reticulocytes of the patients. The exception is Hb Hamilton Hill, which was cloned in expression vectors, causing a significant 25% reduction in the transcriptional activity [53].

This is the first report, to our knowledge, showing a reduction in α-globin mRNA with frameshift mutations in the last exon, indicating that mechanisms other than NMD—probably no-go decay—could be involved in the quality control of the variant mRNAs.

It would be interesting to assess whether other frameshift mutants also display reductions in mRNA, or if those with a more severe phenotype have normal mRNA levels.

## Figures and Tables

**Table 1:** Oligonucleotides used as primers in the reported applications. The sequences and positions of the primers were derived from GeneBank Sequence *NG_000006.1.

Name	Direction, Sequence	Position from the α1 Cap Site	Application	Used with Primer	Amplicon Length (bp)
A For	5′-TGGAGGGTGGAGACGTCCTG-3′	–202/–183	PCR	B Rev	980 bp
B Rev	5′-GGGGGGAGGCCCAAGGGGCAAGAA-3′	+755/+778	PCR		
C For	5′-ACAGGCCACCCTCAACCGTCCT-3′	+183/+204	RE DNA (cod95)	D Rev	352 bp
D Rev	5′-GCAACCCGCGTGATCCTCTGCCCT-3′	+511/+534	RE DNA (cod95)		
E For	5′-GGCAAGAAGGTGGCCGACGC-3′	+332/+351	RE cDNA (cod95)	G Rev	293 bp
F For	5’- TGACCCTCTTCTCTGCACAGCTC-3’	+584/+606	RE DNA (cod109)	G Rev	
G Rev	5’-GAGGCCCAAGGGGCAAGAAGCAT-3’	+751/+773	Seq α1; RT-PCR α1; RE DNA and cDNA		194 bp
H For	5′ -CGCCCTGAGCGACCTGCACGCG-3′	+400/+421	RE cDNA (cod109)	G Rev	230 bp

RE: restriction enzyme digestion; Seq: sequencing analysis; cod: codon.

**Table 2:** Hematological and biochemical data and α-genotype of the family with *Hb Campania*.

Family Relationship	I-1	I-2	II-1	II-2	II-3
Sex/Age (years)	M/56	F/54	F/25	M/22	F/21
RBC (10^12^/L)	4.55	5.16	4.71	5.75	4.55
Hb (g/dL)	13.9	12.7	12.5	13.6	12.5
Ht (L/L)	44.2	41.2	38.8	43.8	38.9
MCV (fL)	97	80	82	76	85
MCH (pg)	30.5	24.6	26.6	23.6	27.5
MCHC (%)	31.4	30.8	32.3	31	32.2
Serum iron (µg/dL)	72	155	80	96	62
Ferritin (ng/mL)	78	315	43	154	10
Transferrin (mg/dL)	370	303	232	276	324
Bil tot (mg/dL)	0.38	0.18	0.14	0.22	0.15
Ret (%)	nor	nor	nor	nor	nor
GOR	− − −	+ + −	− − −	− − −	− − −
Hb A2 (%)	2.7	2.4	2.5	2.3	2.7
Hb F (%)	0.0	0.0	0.0	0.0	0.0
α1 cod95 (−C) carrier	no	yes	no	yes	no

RBC: red blood cells; Hb: hemoglobin; Ht: hematocrit; MCV: mean corpuscular volume; MCH: mean corpuscular hemoglobin; MCHC: mean corpuscular hemoglobin concentration; Bil tot: total bilirubin; Ret: reticulocytes; GOR: globular osmotic resistance.

**Table 3:** Hematological and biochemical data and α-globin genotype of the family with *Hb Sciacca*.

Family	A				B					C			D		E
Parameters	I-1	I-2	II-1	II-2	I-1	II-1	II-2 *	II-2 *	II-3	I-1	I-2	II-1	II-1	II-2	I-1
Sex/Age (yrs)	M/38	F/29	F/09	M/05	M/69	M/39	F/35	F/35	M/32	M/56	F/50	M/31	M/30	F/23	F/21
RBC (10^12^/L)	5.1	5.10	5.30	5.20	4.77	4.76	4.66	5.13	5.07	5.65	5.07	6.30	6.32	5.63	4.62
Hb (g/dL)	16.1	13.2	13.4	13.1	14.6	12.9	12.1	13.4	15.6	15.3	15	16.4	15.1	13.4	12.3
Ht (L/L)	45.4	39.1	41.0	38.4	40.5	37.4	37.0	40.6	44	46.2	44.9	49.5	47.3	41.2	34.6
MCV (fL)	89.3	76.3	77.7	74.2	84.9	78.6	79.4	79.1	86.8	81.8	88.5	78.6	74.8	73.2	75.0
MCH (pg)	31.7	25.8	25.4	25.3	30.6	27.1	26.0	26.1	30.8	27.1	29.6	26.0	23.9	23.8	26.6
MCHC (%)	35.5	33.8	32.7	34.1	36	34.5	32.7	33.0	35.5	33.1	33.4	33.1	31.9	32.5	35.6
Heinz body	nt	nt	nt	nt	nt	nt	nt	absent	nt	nt	nt	nt	nt	nt	nt
Erytho morph	nt	nt	nt	nt	nt	nt	nt	nt	nt	nt	nt	nt	nt	nt	A;P;H
Pink test (%)	nt	nt	nt	nt	nt	nt	nt	3.0	nt	nt	nt	nt	nt	nt	nt
Transferrin (mg/dL)	nt	nt	nt	nt	nt	nt	nt	262	nt	nt	nt	nt	nt	nt	nt
Bilir tot (mg/dL)	nt	nt	nt	nt	nt	nt	nt	0.5	nt	nt	nt	nt	nt	nt	nt
Bilir dir (mg/dL)	nt	nt	nt	nt	nt	nt	nt	0.14	nt	nt	nt	nt	nt	nt	nt
Hapt (mg %)	nt	nt	nt	nt	nt	nt	nt	175	nt	nt	nt	nt	nt	nt	nt
LDH (U/L)	nt	nt	nt	nt	nt	nt	nt	279	nt	nt	nt	nt	nt	nt	nt
Ret (%)	nt	nt	nt	nt	nt	nt	nt	1.04	nt	nt	nt	nt	nt	nt	nt
ZPP (µg/dL)	nt	nt	nt	nt	nt	nt	nt	nt	nt	nt	nt	nt	nt	nt	34
Ferritin (ng/mL)	131	72	56	24	48	102	127	nt	0	113	17	78	67	19	0
Hb A2 (%)	2.9	2.9	3.1	2.9	2.9	2.4	2.3	nt	3	3.1	2.7	2.8	2.7	2.7	2.7
Hb F (%)	0	0	0	0	0	0	0.5	nt	0	0	0	0	0.5	0.5	0.3
α1 cod109 (−C)carrier	no	yes	no	yes	no	yes	yes	yes	no	yes	no	yes	yes	yes	yes

RBC: red blood cells; Hb: hemoglobin; Ht: hematocrit; MCV: mean corpuscular volume; MCH: mean corpuscular hemoglobin; MCHC: mean corpuscular hemoglobin concentration; Erytho morph: erythrocyte morphology; Bilir tot: total bilirubin; Bilir dir: direct bilirubin; Hapt: haptoglobin; LDH: lactate dehydrogenase; Ret: reticulocytes; ZPP: zinc protoporphyrin; A: anisocytosis; P: poikilocytosis; H: hypochromia; nt: not tested; * = same person.

**Table 4:** Frameshift and stop codon mutations in the third exon of the α-globin genes.

Name Current Name HGVS Name	Variant Chain	Stop at Cod	Var %	mRNA %	Genotype–Phenotype Relationship	Abnormal Helices	Ref.
α2 cod90-93 (−8bp); HBA2: c.272_279 delAGCTTCGG	Fs from cod91 and extension of the α-chain from 141 to 170 residues.	170	absent	nt	-SEA/-αCD90-93: HbH disease	FG3–HC3 + 29 aa	[48]
Hb Campania; α1 cod95 (G2) (−C); HBA1: c.286delC or HBA1: c.287delC	α(95)R-S-T-S-S-S-(102)COOH	102	absent	34	Heter: α-thal	G2–G8	p.a.
Hb Lynwood; α2 cod107 (G14) (−T); HBA2: c.323delT	α(107)G-P-W-P-P-T-S-P-P-S-S-P-L-R-C-T-P-P-W-T-S-S-W-L-L-(132)COOH	133	absent	nt	-α3.7/Hb Lynwood: severe α-thal	G14–H15	[49]
Hb Sciacca; α1 cod109 (G16) (−C); HBA1: c.327delC or HBA1: c.328delC	α(109)W-P-P-T-S-P-P-S-S-P-L-R-C-T-P-P-W-T-S-S-W-L-L-(132)COOH	133	absent	15	Heter: α-thal	G16–H15	[2,8,9] p.a.
α1 cod111-115 (−13bp); HBA1: c.333_345delCGCCCACCTCCCC	α(110)A-P-S-S-P-L-R-C-T-P-P-L-R-C-T-P-P-W-T-S-P-W-T-S-S-W-L-L-(128)COOH	128(133)	absent	nt	Heter: α-thal	G17–H11	[43]
α2 cod114 (GH2) (−C); HBA2: c.342delC or HBA2: c.345delC	α(114)P-P-S-S-P-L-R-C-T-P-P-W-T-S-S-W-L-L-(132)COOH	133	absent	nt	-α3.7/α2 cod 113/114–C: severe α-thal, 15% Hb Bart’s	GH2–H15	[50]
α2 cod115 (GH3) (+CC); HBA2: c.343_344insCC or HBA2: c.342-345insCC or HBA2: c.344_345dup	α(114)P-P-P-S-S-P-L-R-C-T-P-P-W-T-S-S-W-L-L-(133)COOH	134(133)	absent	nt	Heter: dominant α-thal	GH3–H16	[51]
α2 cod116 (GH4) GAG>TAG Glu>Stop*; HBA2: c.349G>T	α116(Stop)	116	absent	normal	Heter: α-thal	GH4	[44]
α2 cod116-119 (−11bp); HBA2: c.349_359delGAGTTCACCCC	Fs from cod 115 and extension of the α-chain from 141 to 166 residues.	166	absent	nt	-α3.7/α2 cod116-119 del: Marked microcytosis, Hb H inclusion	GH3–HC3	[52]
α2 cod 127 (H10) GAG>TAG Lys>Stop*; HBA2: c.382A>T	α127(stop)	127	absent	nt	Heter: α-thal	H10	[45]
Hb Hamilton Hill; α2 cod129 (H12) (−C); HBA2: c.388delC	α(129)W, L, L (132)COOH	133	absent	nt; 75 in vitro	Heter: α-thal	H12–H14	[53]
Hb Fez; α1 cod131 (H14) (−T); HBA1: c.396delT	α(131)S-(132)COOH	133	absent	nt	Heter: CHA Very unstable	H14	[54]
Hb Pak Num Po; α1 cod132 (+T); HBA1: c.396_397insT	α(132)C-E-H-R-A-D-L-Q-I-P-L-S-W-S-L-G-G-H-A-S-C-P-L-G-L-P-P-A-P-P-P-L-P-A-P-V-P-P-W-S-L-N-K-(175)V-COOH	175	absent	nt	Heter: α-thal;−SEA/Hb Pak Num Po: tran. dep. HbH	H15–HC3+ 34 aa	[55]
Hb Aalesund; α2 cod133-135 (−7bp); HBA2: c.400_406delAGCACCG	α(133)C-(134)COOH	134(137)	~3 Hb A1c	nt	Heter: balanced hemolytic anemia	H16	[56]
Hb Senlis; α1 cod134 (H17) (−C); HBA1: c.404delC	α(134)T-C-(136)COOH	137	absent	nt	Heter: CHA, very unstable	H17–H18	[54]
Hb Wayne; α2 or α1 cod139 (HC1) (−A); HBA2: c.420delA(or HBA1)	α(139)N-T-V-K-L-E-P-(146)R-COOH	147	12-16	nt	Heter: normal, but absence of a Bohr effect, increased oxygen affinity	HC1–HC3+ 5 aa	[57]

CHA: chronic hemolytic anemia; nt: not tested; pa: present article; td HBH: transfusion-dependent HBH; * = nonsense mutation; Heter: heterozygotes.

## Data Availability

Not applicable.

## Supplementary Materials

The following are available online at https://www.mdpi.com/article/10.3390/biomedicines9101390/s1, Figure S1: 3D model of WT, Hb Campania and Hb Sciacca α-chains, Figure S2: Amino acids sequence and predicted secondary structure of the WT and mutated α-chains, Figure S3:Aanalysis of the mutants HBA1 cod95 (-C) and HBA1 cod109 (-C) with the SIFT software, Figure S4: Analysis of the two mutants HBA1 cod95 (-C) and HBA1 cod109 (-C) with the Mutationtaster software, Figure S5: Donor and acceptor splice site prediction of WT, Hb Campania and Hb Sciacca α1-globin mRNAs, Figure S6: Amino acid sequences of the variant chains HBA1 cod95 (-C) and HBA1 cod109 (-C) from the SIFT bioinformatic tool, Figure S7: Translation output of the normal and mutant HBA1 coding mRNA sequences to a protein sequence using the bioinformatic tool https://web.expasy.org/translate/ (Accessed on 21 June 2021), Figure S8: Amino acids composition of the HBA1 WT, and of HBA1 cod95 (-C) and HBA1 cod109 (-C) by mean of the bioinformatic tool https://web.expasy.org/protparam/ (Accessed on 22 June 2021), Figure S9: Codon usage of HBA1 WT and of HBA1 cod95 (-C) and HBA1 cod109 (-C), my means of the bioinformatic tool http://genomes.urv.es/CAIcal/ (Accessed on 23 June 2021), Figure S10: Codon usage in Homo Sapiens (A), red blood cell (B), and related to the Hemoglobin (C) by means of the Codon Usage Database https://www.kazusa.or.jp/codon/cgi-bin/showcodon.cgi?species=9606 (Accessed on 16 June 2021), Figure S11: Restriction enzyme analysis of DNA amplicomers of the *Hb Campania* and *Hb Sciacca genes*.
