# Supplementary material for: mRNA Analysis of Frameshift Mutations with Stop Codon in the Last Exon: The Case of Hemoglobins Campania [α1 cod95 (−C)] and Sciacca [α1 cod109 (−C)]
Source: Biomedicines. 2021 Oct 4;9(10):1390. doi: 10.3390/biomedicines9101390 (PMC8533187; doi:10.3390/biomedicines9101390)
Supplement: Supplementary file 1 [file biomedicines-09-01390-s001.zip › biomedicines-1346132-supplementary.pdf]

Supplementary materials

mRNA Analysis of Frameshift Mutations with Stop Codon in the Last Exon: The Case of Hemoglobins Campania [α1 cod95 (−C)] and Sciacca [α1 cod109 (−C)]

Giovanna Cardiero[1], Gennaro Musollino[1], Romeo Prezioso[1] and Giuseppina Lacerra [1,*]

[1] Institute of Genetics and Biophysics "Adriano Buzzati Traverso", (IGB-ABT, CNR), National Research Council, 80131 Naples, Italy.

* Correspondence: giuseppina.lacerra@igb.cnr.it;

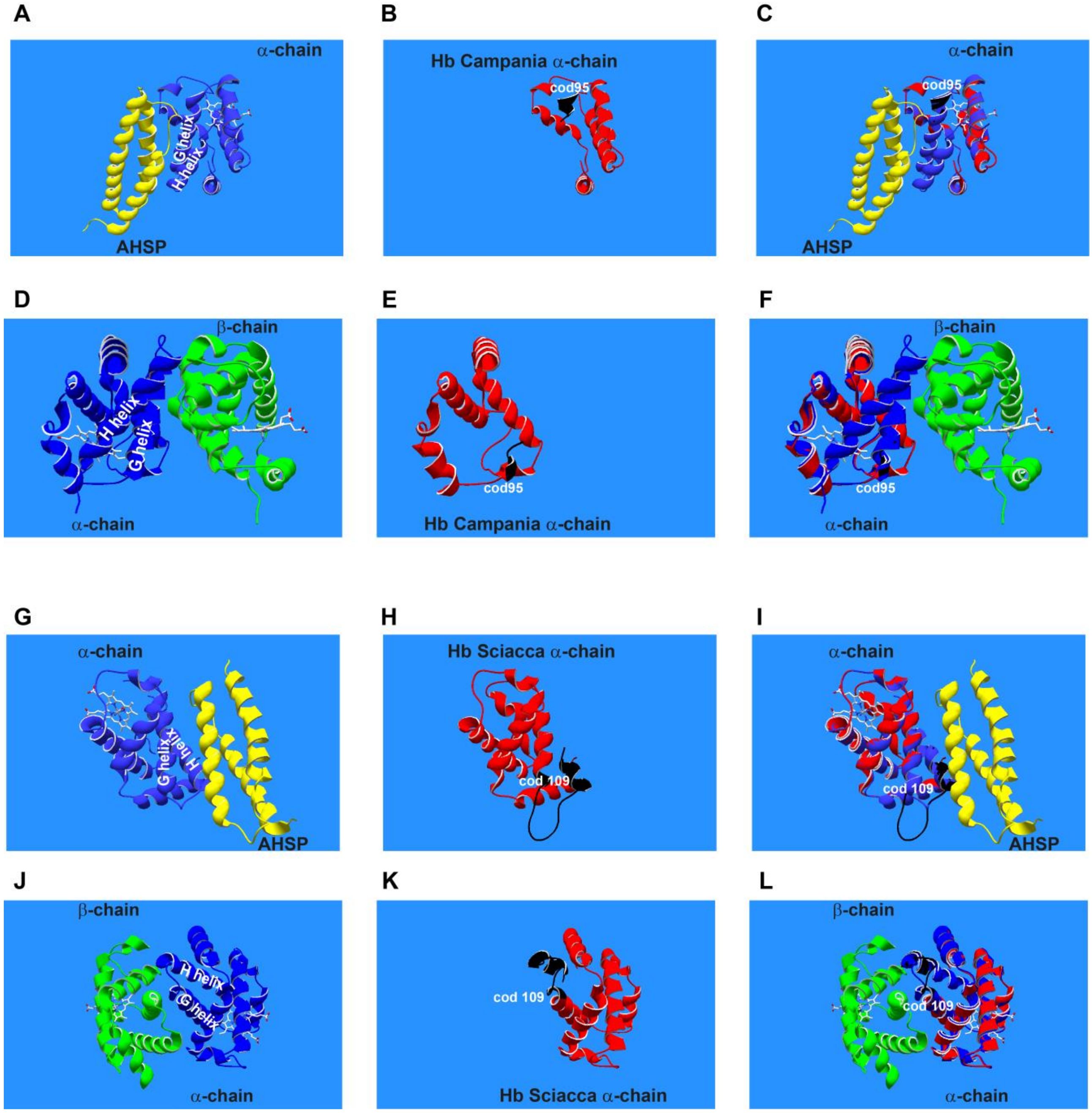

Figure S1. 3D model of WT, Hb Campania and Hb Sciacca α-chains. The 3D structures of the complex between the α-chain and AHSP (PDB code 1Z8U) (A and G) and of the αβ dimer from the structure of the tetrameric human deoxy hemoglobin (PDB code 2HHB) (D and J), respectively. 3D model of the Hb Campania (B, E) and Hb Sciacca (H, K) α-chains obtained using as "template model" the complex α-chain and AHSP (PDB code 1Z8U) and the αβ dimer from the structure of the tetrameric human deoxy hemoglobin (PDB code 2HHB), respectively. The black segments represent the regions involved in the frameshift causing the mutation of 6 aa (Hb Campania) and 23 aa (Hb Sciacca) in the C-terminal region. 3D superimposed model of Hb Campania and Hb Sciacca interacting with AHSP (C, I) and in the Hemoglobin tetramer (F, L). Modelling of the mutants were obtained using the Swiss-PdbViewer.

A) HBA1 WT

```
         10        20        30        40        50        60        70
         |         |         |         |         |         |         |
MVLSPADKTNVKAAWGKVGAHAGEYGAEALERMFLSFPTTKTYFPHFDLSHGSAQVKGHGKKVADALTNA
    hhhhhhhhhhhhh   hhhhhhhhhhhhhhh   hhhh           hhhhhhhhhhhhhhhhh
VAHVDDMPNALSALSDLHAHKLRVDPVNFKLLSHCLLVTLAAHLPAEFTPAVHASLDKFLASVSTVLTSKYR
hh    hhh   hhhhhhhh      hhhhhhhhhhhhhhhh       hhhhhhhhhhhhhhhhhh

            F1-9     FG1-5       G1-19       GH1-5        H1-21        HC1-3
```

B) Hb Campania or HBA1 cod 95 (-C)

```
         10        20        30        40        50        60        70
         |         |         |         |         |         |         |
MVLSPADKTNVKAAWGKVGAHAGEYGAEALERMFLSFPTTKTYFPHFDLSHGSAQVKGHGKKVADALTNA
    hhhhhhhhhhhhh   hhhhhhhhhhhhhhh   hhhh           hhhhhhhhhhhhhhhhh
VAHVDDMPNALSALSDLHAHKLRVDRSTSSS
hh    hhh   hhhhhhhh      hh

            F1-9     FG1-5
```

C) Hb Sciacca or HBA1 cod109 (-C)

```
         10        20        30        40        50        60        70
         |         |         |         |         |         |         |
MVLSPADKTNVKAAWGKVGAHAGEYGAEALERMFLSFPTTKTYFPHFDLSHGSAQVKGHGKKVADALTNA
    hhhhhhhhhhhhh   hhhhhhhhhhhhhhh   hhhh           hhhhhhhhhhhhhhhhh
VAHVDDMPNALSALSDLHAHKLRVDPVNFKLLSHCLLVTWPPTSPPSSPLRCTPPWTSSWLL
hh    hhh   hhhhhhhh      hhhhhhhhhhhhhhh            hhhhhhh

            F1-9     FG1-5       G1-19         GH1-11     H1-7
```

Figure S2. Amino acids sequence (capital letters) and predicted secondary structure (lower-case letters) acquired from the 3D model of the WT (**A**) and mutated α-chains of the human hemoglobin (**B** and **C**). Amino acids mutated due to frameshift and the corresponding positions in the normal α-chain are highlighted with color indicating the type of aminoacids: Red=Polar; Gray=Small non polar; Green=Hydrophobic; Cyan=Aromatics plus cysteine (PSIPRED). Alpha-Helix and non-helix segments of the normal chain are reported in italic letters starting from the cod80, and acquired from ref 28. The alpha-helix and non-helix segments of the Hb Sciacca were acquired from the 3D fitted model using SwissPdbViewer.

	HBA1 cod95 -C	HBA1 cod109 -C
Coordinates	16,177119,177120,1,-/	16,177309,177310,1,-/
Gene ID	ENSG00000206172	ENSG00000206172
Transcript ID	ENST00000320868	ENST00000320868
Substitution Type	FRAMESHIFT	FRAMESHIFT
Region	CDS	CDS
Amino acid position change	95-142	109-142
Effect	damaging	damaging
Confidence Score	0.858	0.858
Classification Path	FS_CP12	FS_CP12
Nucleotide change	GGACC-c-GGTCA	TGACC-c-TGGCC
Amino acid change	KLRVDpvnfkllshcllvtl...(+32 amino acids)...*->KLRVDrstsss*	CLLVTlaahlpaeftpavha...(+18 amino acids)...*->CLLVTwpptsppssplrctp...(+8 amino acids)...*
Indel Location	67%	76%
Causes NMD	NO	NO
Repeat detected		
Transcript visulization	|---{}[]--[.*]--[]{}--->	|---{}[]--[]--[.*]{}--->
Gene Name	HBA1	HBA1
Gene Desc	hemoglobin, alpha 1 [Source:HGNC Symbol;Acc:HGNC:4823]	hemoglobin, alpha 1 [Source:HGNC Symbol;Acc:HGNC:4823]
Protein Family ID	ENSFM00500000269637	ENSFM00500000269637
Protein Family Desc	HEMOGLOBIN SUBUNIT ALPHA	HEMOGLOBIN SUBUNIT ALPHA
Transcript Status	KNOWN	KNOWN
Protein Family Size	5	5
OMIM Disease	HEMOGLOBIN--ALPHA LOCUS 1; HBA1;;3-PRIME @ALPHA-GLOBIN GENE;;MINOR ALPHA-GLOBIN LOCUSMETHEMOGLOBINEMIA, ALPHA-GLOBIN TYPE, INCLUDED;;ERYTHREMIA, ALPHA-GLOBIN TYPE, INCLUDED	HEMOGLOBIN--ALPHA LOCUS 1; HBA1;;3-PRIME @ALPHA-GLOBIN GENE;;MINOR ALPHA-GLOBIN LOCUSMETHEMOGLOBINEMIA, ALPHA-GLOBIN TYPE, INCLUDED;;ERYTHREMIA, ALPHA-GLOBIN TYPE, INCLUDED
1000 Genomes		

Figure S3. Output of the analysis of the mutants HBA1 cod95 (-C) and HBA1 cod109 (-C) with the Sorting Intolerant From Tolerant (SIFT) bioinformatic tool https://sift.bii.a-star.edu.sg/www/SIFT_indels2.html.

Analysis of the mutant HBA1 cod95 (-C) with the Mutationtaster software:	http://www.mutationtaster.org/cgi-bin/MutationTaster/MutationTaster69.cgi?sequence_snippet=TTCGGGTGGACC[C/-]GGTCAACTTCAAG&transcript_stable_id_text=ENST00000320868&gene=HBA1&transcript_stable_id_radio=ENST00000320868&sequence_type=cDNA&nt_alignment=1&alteration_name=HBA1cod95-C.
Analysis of the mutant HBA1 cod109 (-C) with the Mutationtaster software	http://www.mutationtaster.org/cgi-bin/MutationTaster/MutationTaster69.cgi?sequence_snippet=CCTGCTGGTGACC[C/-]TGGCCGCCCA&transcript_stable_id_text=ENST00000320868&nt_alignment=1&gene=HBA1&transcript_stable_id_radio=ENST00000320868&sequence_type=cDNA&alteration_name=HBA1cod109-C

Figure S4. Link of the output of the analysis of the two mutants HBA1 cod95 (-C) and HBA1 cod109 (-C) with the Mutationtaster software.

A) Splice site predictions for 1 sequence with donor score cutoff 0.40, acceptor score cutoff 0.40 (exon/intron boundary shown in larger font):

Donor site predictions for HBA1 WT:

Start	End	Score	Exon Intron
77	91	0.95	gcctggggtaaggtc
126	140	0.89	tggagaggtgaggct
313	327	0.40	tgcccaggttaaggg
448	462	0.99	cttcaaggtgagcgg

Acceptor site predictions for HBA1 WT:

Start	End	Score	Intron Exon
229	269	0.97	tcactctgcttctccccgcaggatgttcctgtccttcccca
299	339	0.67	ctgagccacggctctgcccaggttaagggccacggcaagaa
492	532	0.87	gagatggcgccttcctcgcagggcagaggatcacgcgggtt
583	623	0.95	ctgaccctcttctctgcacagctcctaagccactgcctgct
760	800	0.47	gccccttgggcctcccccagcccctcctccccttcctgca

B) Splice site predictions for 1 sequence with donor score cutoff 0.02, acceptor score cutoff 0.02 (exon/intron boundary shown in larger font):

Donor site predictions for HBA1 WT:

Start	End	Score	Exon Intron
77	91	0.95	gcctggggtaaggtc
107	121	0.08	gagtatggtgcggag
126	140	0.89	tggagaggtgaggct
313	327	0.40	tgcccaggttaaggg
334	348	0.09	caagaaggtggccga
448	462	0.99	cttcaaggtgagcgg
693	707	0.20	ggcttctgtgagcac

Acceptor site predictions for HBA1 WT:

Start	End	Score	Intron Exon
166	206	0.37	ctcgcccgcccggacccacaggccaccctcaaccgtcctgg
229	269	0.97	tcactctgcttctccccgcaggatgttcctgtccttcccca
299	339	0.67	ctgagccacggctctgcccaggttaagggccacggcaagaa
434	474	0.05	gtggacccggtcaacttcaaggtgagcggcgggccgggagc
492	532	0.87	gagatggcgccttcctcgcagggcagaggatcacgcgggtt
583	623	0.95	ctgaccctcttctctgcacagctcctaagccactgcctgct
760	800	0.47	gccccttgggcctcccccagcccctcctccccttcctgca

C) Splice site predictions for 2 sequences with donor score cutoff 0.02, acceptor score cutoff 0.02 (exon/intron boundary shown in larger font):

Acceptor site predictions for HBA1 cod 95 (-C):

Start	End	Score	Intron Exon
433	473	0.03	ggtggaccggtcaacttcaaggtgagcggcgggccgggagc

D) Splice site predictions for 1 sequence with donor score cutoff 0.02, acceptor score cutoff 0.02 (exon/intron boundary shown in larger font):

Donor site predictions for HBA1 cod 109 (-C):

Start	End	Score	Exon Intron
618	632	0.02	cctgctggtgacctg

Figure S5. Donor and acceptor splice site prediction of WT, Hb Campania and Hb Sciacca α[illegible]-globin mRNA. Donor and acceptor splice site prediction for HBA1 WT (NM_000558.5) with score cutoff 0,40 (**A**) and 0.02 (**B**). The analysis of the splice site prediction for the HBA1 cod95 (-C) and HBA1 cod109 (-C) showed only variation with score cutoff 0.02 respectively for the reported Acceptor site (**C**) and Donor site (**D**). The position of the two mutations are underlined.

HBA1 cod95 (-C)

```
HBA1 WT >ENST00000320868; MISMATCH = 95-142
MVLSPADKTNVKAAWGKVGAHAGEYGAEALERMFLSFPTTKTYFPHFDLSHGSAQVKGHGKKVADALTNAVAHVDDMPNA
LSALSDLHAHKLRVDpvnfkllshcllvtlaahlpaeftpavhasldkflasvstvltskyr

HBA1 cod95 (-C) >ENST00000320868; MISMATCH = 95-101
MVLSPADKTNVKAAWGKVGAHAGEYGAEALERMFLSFPTTKTYFPHFDLSHGSAQVKGHGKKVADALTNAVAHVDDMPNA
LSALSDLHAHKLRVDrstsss
```

HBA1 cod109 (-C)

```
HBA1 WT >ENST00000320868; MISMATCH = 109-142
MVLSPADKTNVKAAWGKVGAHAGEYGAEALERMFLSFPTTKTYFPHFDLSHGSAQVKGHGKKVADALTNAVAHVDDMPNA
LSALSDLHAHKLRVDPVNFKLLSHCLLVTlaahlpaeftpavhasldkflasvstvltskyr

HBA1 cod109 (-C) >ENST00000320868; MISMATCH = 109-132
MVLSPADKTNVKAAWGKVGAHAGEYGAEALERMFLSFPTTKTYFPHFDLSHGSAQVKGHGKKVADALTNAVAHVDDMPNA
LSALSDLHAHKLRVDPVNFKLLSHCLLVTwpptsppssplrctppwtsswll
```

Figure S6. Amino acid sequences of the variant chains HBA1 cod95 (-C) and HBA1 cod109 (-C) from the SIFT bioinformatic tool https://sift.bii.a-star.edu.sg/www/SIFT_indels2.html. In black the conserved region, in red the mutated region in the variant and normal chains.

HBA1 WT

```
atg gtg ctg tct cct gcc gac aag acc aac gtc aag gcc gcc tgg ggt aag gtc ggc gcg
 M   V   L   S   P   A   D   K   T   N   V   K   A   A   W   G   K   V   G   A
cac gct ggc gag tat ggt gcg gag gcc ctg gag agg atg ttc ctg tcc ttc ccc acc acc
 H   A   G   E   Y   G   A   E   A   L   E   R   M   F   L   S   F   P   T   T
aag acc tac ttc ccg cac ttc gac ctg agc cac ggc tct gcc cag gtt aag ggc cac ggc
 K   T   Y   F   P   H   F   D   L   S   H   G   S   A   Q   V   K   G   H   G
aag aag gtg gcc gac gcg ctg acc aac gcc gtg gcg cac gtg gac gac atg ccc aac gcg
 K   K   V   A   D   A   L   T   N   A   V   A   H   V   D   D   M   P   N   A
ctg tcc gcc ctg agc gac ctg cac gcg cac aag ctt cgg gtg gac ccg gtc aac ttc aag
 L   S   A   L   S   D   L   H   A   H   K   L   R   V   D   P   V   N   F   K
ctc cta agc cac tgc ctg ctg gtg acc ctg gcc gcc cac ctc ccc gcc gag ttc acc cct
 L   L   S   H   C   L   L   V   T   L   A   A   H   L   P   A   E   F   T   P
gcg gtg cac gcc tcc ctg gac aag ttc ctg gct tct gtg agc acc gtg ctg acc tcc aaa
 A   V   H   A   S   L   D   K   F   L   A   S   V   S   T   V   L   T   S   K
tac cgt taa
 Y   R   -
```

HBA1 cod95 (-C)

```
atg gtg ctg tct cct gcc gac aag acc aac gtc aag gcc gcc tgg ggt aag gtc ggc gcg
 M   V   L   S   P   A   D   K   T   N   V   K   A   A   W   G   K   V   G   A
cac gct ggc gag tat ggt gcg gag gcc ctg gag agg atg ttc ctg tcc ttc ccc acc acc
 H   A   G   E   Y   G   A   E   A   L   E   R   M   F   L   S   F   P   T   T
aag acc tac ttc ccg cac ttc gac ctg agc cac ggc tct gcc cag gtt aag ggc cac ggc
 K   T   Y   F   P   H   F   D   L   S   H   G   S   A   Q   V   K   G   H   G
aag aag gtg gcc gac gcg ctg acc aac gcc gtg gcg cac gtg gac gac atg ccc aac gcg
 K   K   V   A   D   A   L   T   N   A   V   A   H   V   D   D   M   P   N   A
ctg tcc gcc ctg agc gac ctg cac gcg cac aag ctt cgg gtg gac cgg tca act tca agc
 L   S   A   L   S   D   L   H   A   H   K   L   R   V   D   R   S   T   S   S
tcc taa
 S   -
```

HBA1 cod109 (-C)

```
atg gtg ctg tct cct gcc gac aag acc aac gtc aag gcc gcc tgg ggt aag gtc ggc gcg
 M   V   L   S   P   A   D   K   T   N   V   K   A   A   W   G   K   V   G   A
cac gct ggc gag tat ggt gcg gag gcc ctg gag agg atg ttc ctg tcc ttc ccc acc acc
 H   A   G   E   Y   G   A   E   A   L   E   R   M   F   L   S   F   P   T   T
aag acc tac ttc ccg cac ttc gac ctg agc cac ggc tct gcc cag gtt aag ggc cac ggc
 K   T   Y   F   P   H   F   D   L   S   H   G   S   A   Q   V   K   G   H   G
aag aag gtg gcc gac gcg ctg acc aac gcc gtg gcg cac gtg gac gac atg ccc aac gcg
 K   K   V   A   D   A   L   T   N   A   V   A   H   V   D   D   M   P   N   A
ctg tcc gcc ctg agc gac ctg cac gcg cac aag ctt cgg gtg gac ccg gtc aac ttc aag
 L   S   A   L   S   D   L   H   A   H   K   L   R   V   D   P   V   N   F   K
ctc cta agc cac tgc ctg ctg gtg acc tgg ccg ccc acc tcc ccg ccg agt tca ccc ctg
 L   L   S   H   C   L   L   V   T   W   P   P   T   S   P   P   S   S   P   L
cgg tgc acg cct ccc tgg aca agt tcc tgg ctt ctg tga
 R   C   T   P   P   W   T   S   S   W   L   L   -
```

Figure S7. Translation output of the normal and mutant HBA1 coding mRNA sequences to a protein sequence using the bioinformatic tool https://web.expasy.org/translate/. The differences in the amino acids composition are highlighted in gray in the two variants.

HBA1 WT

Ala	(A)	21	14.8%
Arg	(R)	3	2.1%
Asn	(N)	4	2.8%
Asp	(D)	8	5.6%
Cys	(C)	1	0.7%
Gln	(Q)	1	0.7%
Glu	(E)	4	2.8%
Gly	(G)	7	4.9%
His	(H)	10	7.0%
Ile	(I)	0	0.0%
Leu	(L)	18	12.7%
Lys	(K)	11	7.7%
Met	(M)	3	2.1%
Phe	(F)	7	4.9%
Pro	(P)	7	4.9%
Ser	(S)	11	7.7%
Thr	(T)	9	6.3%
Trp	(W)	1	0.7%
Tyr	(Y)	3	2.1%
Val	(V)	13	9.2%
Pyl	(O)	0	0.0%
Sec	(U)	0	0.0%

HBA1 cod95 (-C)

Ala	(A)	15	14.9%
Arg	(R)	3	3.0%
Asn	(N)	3	3.0%
Asp	(D)	7	6.9%
Cys	(C)	0	0.0%
Gln	(Q)	1	1.0%
Glu	(E)	3	3.0%
Gly	(G)	7	6.9%
His	(H)	7	6.9%
Ile	(I)	0	0.0%
Leu	(L)	9	8.9%
Lys	(K)	8	7.9%
Met	(M)	3	3.0%
Phe	(F)	4	4.0%
Pro	(P)	4	4.0%
Ser	(S)	10	9.9%
Thr	(T)	6	5.9%
Trp	(W)	1	1.0%
Tyr	(Y)	2	2.0%
Val	(V)	8	7.9%
Pyl	(O)	0	0.0%
Sec	(U)	0	0.0%

HBA1 cod109 (-C)

Ala	(A)	15	11.4%
Arg	(R)	3	2.3%
Asn	(N)	4	3.0%
Asp	(D)	7	5.3%
**Cys**	**(C)**	**2**	**1.5%**
Gln	(Q)	1	0.8%
Glu	(E)	3	2.3%
Gly	(G)	7	5.3%
His	(H)	8	6.1%
Ile	(I)	0	0.0%
Leu	(L)	16	12.1%
Lys	(K)	9	6.8%
Met	(M)	3	2.3%
Phe	(F)	5	3.8%
**Pro**	**(P)**	**12**	**9.1%**
**Ser**	**(S)**	**12**	**9.1%**
Thr	(T)	9	6.8%
**Trp**	**(W)**	**4**	**3.0%**
Tyr	(Y)	2	1.5%
Val	(V)	10	7.6%
Pyl	(O)	0	0.0%
Sec	(U)	0	0.0%

Figure S8. Comparison of the amino acids composition of the HBA1 WT, and of the two frameshift mutants HBA1 cod95 (-C) and HBA1 cod109 (-C) calculated by mean of the bioinformatic tool https://web.expasy.org/protparam/. The amino acids showing an increase in number have been reported in bold.

CODONS	Aminoacids	HBA1 WT	HBA1 cod95 (-C)	HBA1 cod109 (-C)
TTT	F	0	0	0
TTC	F	7	4	5
TTA	L	0	0	0
TTG	L	0	0	0
CTT	L	1	1	2
CTC	L	2	0	1
CTA	L	1	0	1
CTG	L	14	8	12
ATT	I	0	0	0
ATC	I	0	0	0
ATA	I	0	0	0
GTT	V	1	1	1
GTC	V	3	2	3
GTA	V	0	0	0
GTG	V	9	5	6
TCT	S	3	2	2
TCC	S	4	3	4
TCA	S	0	2	1
TCG	S	0	0	0
AGT	S	0	0	2
AGC	S	4	3	3
CCT	P	2	1	2
CCC	P	3	2	5
CCA	P	0	0	0
CCG	P	2	1	5
ACT	T	0	1	0
ACC	T	9	5	7
ACA	T	0	0	1
ACG	T	0	0	1
GCT	A	2	1	1
GCC	A	12	8	8
GCA	A	0	0	0
GCG	A	7	6	6
TAT	Y	1	1	1
TAC	Y	2	1	1
CAT	H	0	0	0
CAC	H	10	7	8
CAA	Q	0	0	0
CAG	Q	1	1	1
AAT	N	0	0	0
AAC	N	4	3	4
AAA	K	1	0	0
AAG	K	10	8	9
GAT	D	0	0	0
GAC	D	8	7	7
GAA	E	0	0	0
GAG	E	4	3	3
TGT	C	0	0	0
TGC	C	1	0	2
CGT	R	1	0	0
CGC	R	0	0	0
CGA	R	0	0	0
CGG	R	1	2	2
AGA	R	0	0	0
AGG	R	1	1	1
GGT	G	2	2	2
GGC	G	5	5	5
GGA	G	0	0	0
GGG	G	0	0	0
ATG	M	3	3	3
TGG	W	1	1	4

Figure S9. Comparison of the codon usage of HBA1 WT and of the two mutants HBA1 cod95 (-C) and HBA1 cod109 (-C), obtained my means of the bioinformatic tool http://genomes.urv.es/CAIcal/. The new codons or increased in number have been highlighted with different background colors.

(A) Homo sapiens **[gbpri]: 93487 CDS's (40662582 codons)**

fields: [triplet] [amino acid] [fraction] [frequency: per thousand] ([number])

UUU F 0.46 17.6 (714298)	UCU S 0.19 15.2 (618711)	UAU Y 0.44 12.2 (495699)	UGU C 0.46 10.6 (430311)
UUC F 0.54 20.3 (824692)	UCC S 0.22 17.7 (718892)	UAC Y 0.56 15.3 (622407)	UGC C 0.54 12.6 (513028)
UUA L 0.08 7.7 (311881)	UCA S 0.15 12.2 (496448)	UAA * 0.30 1.0 (40285)	UGA * 0.47 1.6 (63237)
UUG L 0.13 12.9 (525688)	UCG S 0.05 4.4 (179419)	UAG * 0.24 0.8 (32109)	UGG W 1.00 13.2 (535595)
CUU L 0.13 13.2 (536515)	CCU P 0.29 17.5 (713233)	CAU H 0.42 10.9 (441711)	CGU R 0.08 4.5 (184609)
CUC L 0.20 19.6 (796638)	CCC P 0.32 19.8 (804620)	CAC H 0.58 15.1 (613713)	CGC R 0.18 10.4 (423516)
CUA L 0.07 7.2 (290751)	CCA P 0.28 16.9 (688038)	CAA Q 0.27 12.3 (501911)	CGA R 0.11 6.2 (250760)
CUG L 0.40 39.6 (1611801)	CCG P 0.11 6.9 (281570)§	CAG Q 0.73 34.2 (1391973)	CGG R 0.20 11.4 (464485)
AUU I 0.36 16.0 (650473)	ACU T 0.25 13.1 (533609)	AAU N 0.47 17.0 (689701)	AGU S 0.15 12.1 (493429)
AUC I 0.47 20.8 (846466)	ACC T 0.36 18.9 (768147)	AAC N 0.53 19.1 (776603)	AGC S 0.24 19.5 (791383)
AUA I 0.17 7.5 (304565)	ACA T 0.28 15.1 (614523)	AAA K 0.43 24.4 (993621)	AGA R 0.21 12.2 (494682)
AUG M 1.00 22.0 (896005)	ACG T 0.11 6.1 (246105)§	AAG K 0.57 31.9 (1295568)	AGG R 0.21 12.0 (486463)
GUU V 0.18 11.0 (448607)	GCU A 0.27 18.4 (750096)	GAU D 0.46 21.8 (885429)	GGU G 0.16 10.8 (437126)
GUC V 0.24 14.5 (588138)	GCC A 0.40 27.7 (1127679)	GAC D 0.54 25.1 (1020595)	GGC G 0.34 22.2 (903565)
GUA V 0.12 7.1 (287712)	GCA A 0.23 15.8 (643471)	GAA E 0.42 29.0 (1177632)	GGA G 0.25 16.5 (669873)
GUG V 0.46 28.1 (1143534)	GCG A 0.11 7.4 (299495)	GAG E 0.58 39.6 (1609975)	GGG G 0.25 16.5 (669768)

Coding GC 52.27% 1st letter GC 55.72% 2nd letter GC 42.54% 3rd letter GC 58.55%

(B) Homo sapiens **"red blood cell" 2 CDS's (486 codons)**

fields: [triplet] [frequency: **per thousand**] ([number])

UUU 22.6(11)	UCU 8.2(4)	UAU 14.4(7)	UGU 14.4(7)	UUC 22.6(11)	UCC 20.6(10)
UAC 4.1(2)	UGC 2.1(1)§	UUA 2.1(1)	UCA 16.5(8)	UAA 4.1(2)	UGA 0.0(0)
UUG 24.7(12)	UCG 2.1(1)	UAG 0.0(0)	UGG 12.3(6)	CUU 6.2(3)§	CCU 35.0(17)
CAU 14.4(7)	CGU 6.2(3)	CUC 8.2(4)	CCC 12.3(6)	CAC 12.3(6)	CGC 0.0(0)
CUA 6.2(3)	CCA 18.5(9)	CAA 20.6(10)	CGA 2.1(1)	CUG 24.7(12)	CCG 6.2(3)§
CAG 28.8(14)	CGG 4.1(2)§	AUU 32.9(16)	ACU 20.6(10)	AAU 24.7(12)	AGU 16.5(8)
AUC 20.6(10)	ACC 20.6(10)	AAC 12.3(6)	AGC 16.5(8)	AUA 14.4(7)	ACA 18.5(9)
AAA 80.2(39)	AGA 12.3(6)	AUG 12.3(6)	ACG 4.1(2)§	AAG 47.3(23)	AGG 12.3(6)
GUU 12.3(6)	GCU 6.2(3)	GAU 41.2(20)	GGU 18.5(9)	GUC 16.5(8)	GCC 14.4(7)
GAC 20.6(10)	GGC 10.3(5)	GUA 12.3(6)	GCA 22.6(11)	GAA 26.7(13)	GGA 16.5(8)
GUG 6.2(3)	GCG 0.0(0)	GAG 26.7(13)	GGG 6.2(3)		

Coding GC 42.39% 1st letter GC 46.30% 2nd letter GC 37.65% 3rd letter GC 43.21%

(C) *Homo sapiens* "Hemoglobin" 58 CDS's (9770 codons)

fields: [triplet] [frequency: **per thousand**] ([number])

UUU 13.4(131)	UCU 13.0(127)	UAU 5.6(55)	UGU 6.8(66)	UUC 33.9(331)	UCC 23.1(226)
UAC 16.2(158)	UGC 8.6(84)	UUA 1.8(18)	UCA 2.4(23)§	UAA 2.6(25)	UGA 2.9(28)
UUG 6.9(67)	UCG 3.3(32)	UAG 0.4(4)	UGG 12.5(122)	CUU 5.9(58)	CCU 14.3(140)
CAU 10.2(100)	CGU 4.9(48)	CUC 20.0(195)	CCC 14.1(138)	CAC 39.1(382)	CGC 10.3(101)
CUA 4.4(43)	CCA 8.3(81)	CAA 3.2(31)	CGA 3.3(32)	CUG 79.7(779)	CCG 9.4(92)
CAG 25.5(249)	CGG 8.5(83)	AUU 5.8(57)	ACU 11.0(107)	AAU 7.7(75)	AGU 8.0(78)
AUC 14.5(142)	ACC 34.2(334)	AAC 21.6(211)	AGC 19.3(189)	AUA 2.9(28)	ACA 5.8(57)§
AAA 10.5(103)	AGA 6.0(59)	AUG 19.3(189)	ACG 4.5(44)§	AAG 51.3(501)	AGG 12.6(123)
GUU 11.2(109)	GCU 20.4(199)	GAU 15.8(154)	GGU 12.1(118)	GUC 17.6(172)	GCC 53.1(519)
GAC 34.3(335)	GGC 33.7(329)	GUA 3.3(32)	GCA 8.5(83)	GAA 14.4(141)	GGA 8.6(84)
GUG 54.9(536)	GCG 21.1(206)	GAG 34.7(339)	GGG 7.0(68)		

Coding GC 58.94% 1st letter GC 61.17% 2nd letter GC 41.15% 3rd letter GC 74.51%

Figure S10. Codon usage in Homo Sapiens (A), red blood cell (B), and related to the Hemoglobin (C) obtained by means of the Codon Usage Database https://www.kazusa.or.jp/codon/cgi-bin/showcodon.cgi?species=9606. The codons that have increased in the variant globins mRNA HBA1 cod95 (-C) and HBA1 cod109 (-C) have been highlighted with the same background colors used in Figure S7. § indicate the codon used with very low frequencies (< 7%).

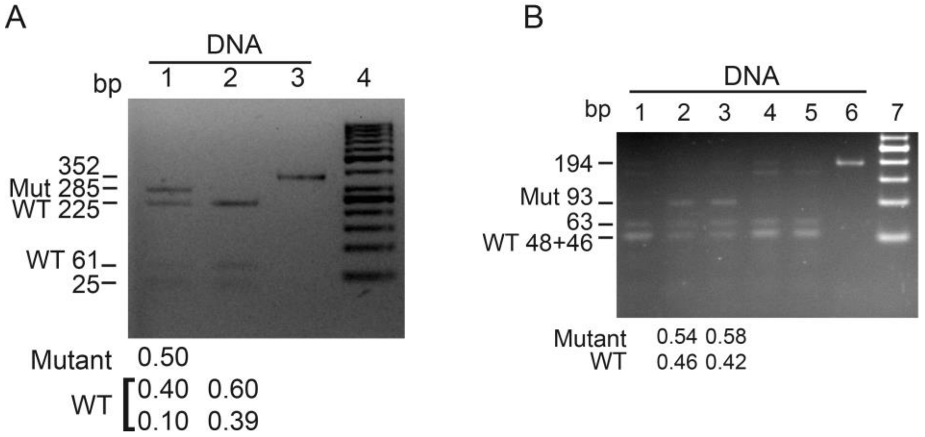

Figure S11. Restriction enzyme analysis of DNA amplicomers of the *Hb Campania* and *Hb Sciacca genes*. (**A**) DNA amplicomers of 352 bp, digested by the restriction enzyme NlaIV, and separated on a 3.5% NuSieve 3:1 agarose gel. Lane 1: DNA of the Hb Campania carrier; Lane 2: DNA of the control subject; Lane 3: undigested sample; Lane 4: 50 bp ladder. The fragments' lengths are reported on the left. The Hb Campania eliminates the NlaIV restriction site GGA'CCC, generating an anomalous longer band of 285 bp, corresponding to the sum of the two WT-specific bands (225 and 61 bp for DNA), minus the deleted cytidine base. The relative amounts of the longer abnormal bands and the WT-bands are reported in the lower section. (**B**) DNA amplicomers of 194 bp digested with the restriction enzyme BseDI and separated on a 3.5% NuSieve 3:1 agarose gel. Lanes 1 and 4: DNA of subjects with WT α1-globin; Lanes 2 and 3: DNA of the Hb Sciacca heterozygotes; Lane 5: DNA of the control subject; Lane 6: undigested sample; Lane 7: 50 bp ladder. The Hb Sciacca eliminates the BseDI restriction site C'CCTGG, generating an anomalous longer band of 93 bp, corresponding respectively to the sum of the two WT-specific bands (48 and 46 bp for DNA), minus the deleted cytidine base. The fragments' lengths are reported on the left. The relative quantities of longer abnormal bands and WT bands are reported in the lower section.
